# Leveraging Viscosity to Unlock the Osteogenic Potential of BMP‐2 Mimetic DWIVA

**DOI:** 10.1002/smll.202508366

**Published:** 2025-12-30

**Authors:** Finlay Cunniffe, Ziyuan Luo, Eva Barcelona‐Estaje, Eva Forrest, Manuel Salmeron‐Sanchez, Marco Cantini

**Affiliations:** ^1^ Centre for the Cellular Microenvironment Advanced Research Centre University of Glasgow Glasgow UK; ^2^ Division of Biomedical Engineering James Watt School of Engineering University of Glasgow Glasgow UK; ^3^ Institute for Bioengineering of Catalonia (IBEC) The Barcelona Institute for Science and Technology (BIST) Barcelona Spain; ^4^ Institució Catalana de Recerca i Estudis Avançats (ICREA) Barcelona Spain

**Keywords:** BMP‐2, DWIVA, mesenchymal stem cell, osteogenesis, viscosity

## Abstract

Growth factor mimetics offer great potential for osteogenic biomaterials; yet, their use remains limited, likely due to an incomplete understanding of the effects of the microenvironment on their activity. The extracellular matrices (ECMs) where growth factors are presented in vivo are viscoelastic environments, where dynamic receptor‐ligand interactions drive cellular responses. Here, supported lipid bilayers of varying viscosity are used as 2D dynamic ECM models, where the bone morphogenetic 2 (BMP‐2) mimetic DWIVA is presented to mesenchymal stem cells alongside the adhesive peptide RGD. DWIVA is demonstrated to have no impact on mechanotransductive processes, including actin organisation, focal adhesion formation and YAP localisation, which are exclusively controlled by viscosity via RGD. Interestingly, DWIVA promotes osteogenic markers’ expression only on a viscous bilayer, through a process that involves non‐canonical BMP‐2 pathways; on a mobile bilayer or on a static control, it lacks osteogenic activity. Crucially, osteogenesis is accompanied by a translocation of BMP receptor 1a to the cell edge, where it colocalises with focal adhesions. Our ECM models hence reveal that both a viscosity‐enabled threshold of cell‐generated forces and a dynamic environment are necessary to harness the osteogenic potential of DWIVA, uncovering key microenvironment properties for the design of DWIVA‐based biomaterials.

## Graphical abstract

Dynamic interfaces based on supported lipid bilayers demonstrate that the osteogenic potential of the BMP‐2 mimetic DWIVA can be harnessed by modulating substrate viscosity. This work highlights that the viscous properties of the cellular microenvironment play a crucial role in the mechanosensitive regulation of growth factor activity.

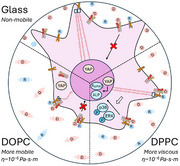

## 1 Introduction

The design of biomaterials requires a deep understanding of how cells respond to their extracellular environment during complex biological processes, whether it be to recapitulate tissues in vitro or for tissue regeneration [1, 2]. In vivo, tissues exhibit different mechanical properties due to the presence of a variety of macromolecules and cells within the extracellular matrix (ECM). These properties are known to control cell behaviour: the elasticity of a material, for example, can guide the differentiation of mesenchymal stem cells (MSCs) toward specific lineages in the absence of other stimuli, with osteogenic differentiation being favoured at higher elasticities [3]. Crucially, the ECM is a highly dynamic environment that displays viscoelastic properties. The availability and access of cells to ligands at the nano and microscale – adhesive proteins, growth factors, other cells – can change over time in both homeostasis and disease, as components are reorganised and new macromolecules are deposited by cells [4, 5]. However, many of the current biomaterial platforms do not take into account the true complexity of native tissues; this ultimately limits their performance in promoting tissue regeneration and their applicability in clinics [2, 6]. It is therefore imperative to understand the mechanisms underlying cellular responses to the great diversity of ECM stimuli, in a bid to improve biomaterial design.

One of the fundamental cellular responses that later influences cell fate is the cell's adhesive response to its surroundings. This is mediated through a process governed by the so‐called molecular clutch. Specifically, cell surface integrin receptors bind to ECM adhesive ligands (such as fibronectin‐derived RGD) extracellularly, as well as to the cytoskeleton intracellularly via adaptor proteins such as talin and vinculin [7, 8]. Integrin binding to ECM ligands causes cells to exert a force on the extracellular environment; sufficient force, for example as a result of a substrate's high elasticity, results in the stabilisation of the integrin‐actin interaction, as vinculin binds to unfolded talin, actin stress fibres are formed, and more integrins are recruited to the site allowing stabilisation and reinforcement of the resulting focal adhesions (FAs) [7, 9]. The signalling cascade that ensues as a result of clutch engagement allows nuclear shuttling of transcription factors such as Yes‐associated protein 1 (YAP), thereby offering a method through which mechanical properties of the ECM can directly alter cell behaviours and fate through changes in gene transcription (mechanotransduction) [10, 11]. Much of the work investigating the role of the molecular clutch has been carried out on more typically ‘elastic’ environments, but tissues in the body – including bone – are viscoelastic; hence, introducing a time‐dependent component to biomaterials enables improved recapitulation of the native environments experienced by cells [12]. For example, increased stress relaxation rates in hydrogels have been shown to lead to greater osteogenic differentiation of both MSCs and MSC spheroids [13, 14]. There are, however, a plethora of methods for controlling and conferring viscosity to biomaterials, and these can affect how dynamic a given system is in terms of ligands’ availability and ability to be reorganised, influencing how well cells spread and remodel their environment [12]. There is therefore a need to untangle the properties of these substrates and understand cell responses at a more fundamental level through the uncoupling of ‘elastic’ and ‘viscous’ properties, so that current viscoelastic biomaterials can be improved. To this end, supported lipid bilayers (SLBs) are a dynamic cell culture platform that offers a unique method for exploring cell responses to viscosity in the absence of elastic cues [15, 16]. Indeed, we have previously developed RGD‐functionalised SLBs from two lipids that differ in their melting transition temperatures (T_m_); SLBs made from DOPC (1,2‐dioleoyl‐sn‐glycero‐3‐phosphocholine, with a transition temperature of −17°C) are more mobile at cell culture conditions, while DPPC (1,2‐dipalmitoyl‐sn‐glycero‐3‐phosphocholine, T_m_ = 42°C) is more viscous. Using these SLBs, we previously demonstrated that viscosity regulates the molecular clutch and consequently the activation of mechanotransductive pathways [17, 18].

While substrate dynamic mechanical properties can regulate cell adhesive responses through cell‐ECM interactions, other biochemical factors from the ECM, such as growth factors (GFs), have a major impact on cell behaviour; as such, their controlled presentation has further potential to improve the design of biomaterials. Indeed, the presentation and delivery method of growth factors from biomaterials can alter their biological activity through an interplay between mechanotransduction and GF signalling [19]. In the context of bone engineering, BMP‐2 is the most studied and the most commonly used GF, and was the first to be clinically approved [20, 21]. GF binding results in the oligomerisation of BMP receptors Ia (BMPRIa) and II, which either stimulate osteogenesis directly through activation of canonical Smad signalling, or through a variety of non‐Smad pathways within the cell [22]. While scaffold coating and soluble delivery have shown great success in promoting osteogenesis in research and clinically, the use of BMP‐2 can often be accompanied by severe side effects owing to high doses and off‐target effects [21, 23]. Biomaterials that incorporate immobilised BMP‐2 have hence been developed to improve its efficacy at lower doses compared to soluble delivery; this solid‐phase presentation of BMP‐2 better mimics how growth factors are presented to cells natively in the ECM [19, 24]. Crucially, the activity of BMP‐2 has also been tightly linked to the mechanical properties of the environment where it is presented; indeed, a threshold of elasticity has been shown to exist below which the GF has no osteogenic effect [25, 26], and a mechanically complex environment which mimics the heterogeneity of natural ECMs has been seen to facilitate BMP‐2 signalling [27].

A further refinement in biomaterial design is the use of mimetic peptides derived from the active site of a particular GF. These mimetic peptides are cheaper and more stable than the full‐length proteins, and allow ease of functionalisation and increased control over the designed biomaterial systems, ultimately offering the potential for a more consistent differentiation of MSCs [28]. Multiple peptides derived from BMP‐2 have been investigated for their osteoinductive potential, and one that remains promising yet understudied is known as DWIVA, an amino acid sequence (Aspartic acid, Tryptophan, Isoleucine, Valine, Alanine) derived from residues 30‐34 of the ‘wrist’ epitope of BMP‐2 [29]. This peptide has been shown to elicit osteogenesis via non‐canonical BMP‐2 pathways in both MSCs and other cell lines, and has been used to functionalise pro‐osteogenic microgels [30, 31, 32, 33]. Its activity, however, is dependent on other environmental factors. One study that used functionalised elastic hydrogels with random incorporation of DWIVA found no osteogenic activity from the peptide [34], while there were also few differences observed in the osteogenic activity of microgels functionalised with either DWIVA or a scrambled‐DWIVA peptide [32]. Recent research using RGD/DWIVA bimimetic peptides has suggested that the activity of DWIVA may be linked to the synergistic activation of cytoskeletal machinery [30, 35]. However, studies into the interplay between adhesive mechanotransductive responses and BMP‐2‐ or DWIVA‐signalling have been based on a traditional mechanically static interpretation of the ECM and of ECM mimetics, and their dynamic behaviour has been largely ignored. A further understanding of the mechanisms underlying these cell responses in a dynamic system is therefore needed to enable the optimisation of more complex biomaterials with improved osteogenic efficacy and potential. Within this perspective, SLBs offer again a suitable platform, as their ease of functionalisation with different types of ligands allows for investigating the cooperation or competition between them in a dynamic setting [18].

Based on these considerations, in this work, we set out to explore the contribution of viscosity to the regulation of GF activity. Indeed, while native ECMs are complex viscoelastic systems, isolating the role of the viscous part of viscoelasticity highlights its biological relevance, informing the design of ECM mimetics. We have used SLBs with varying viscosity as dynamic, purely viscous cell culture models to elucidate the mechanisms that underpin the interplay between ECM dynamics and GF activity. To this end, we functionalised DOPC and DPPC SLBs with RGD or a mixture of RGD and DWIVA, using glass as a non‐mobile control. We demonstrate that the presentation of matrix‐bound DWIVA does not affect adhesion‐based mechanotransductive pathways of adhering MSCs, with cell spreading, FA formation and YAP nuclear translocation increasing with substrate viscosity independently of functionalisation. However, we critically show that DWIVA elicits GF signalling through MAPK p38 and ERK1/2 pathways and supports osteogenic differentiation only on viscous DPPC, with no GF activity seen instead on either mobile DOPC or non‐mobile glass. Critically, only SLBs, due to their dynamic nature, are seen to allow adhesive and GF receptors to co‐localise. Hence, we believe that a dynamic environment and a minimal threshold of cell‐generated mechanical forces, as supported only by DPPC SLBs, are both necessary to harness DWIVA's osteogenic potential. Taken together, our results demonstrate that ECM dynamics can be leveraged to harness the osteoinductive potential of the DWIVA GF‐mimetic peptide through non‐canonical MAPK BMP‐2 pathways and highlight the need to recapitulate the dynamic nature of native ECMs for an efficient design of osteogenic biomaterials.

## 2 Results and Discussion

### 2.1 DWIVA has no Effect on Viscosity‐Dependent Cell Morphology

**FIGURE 1:**
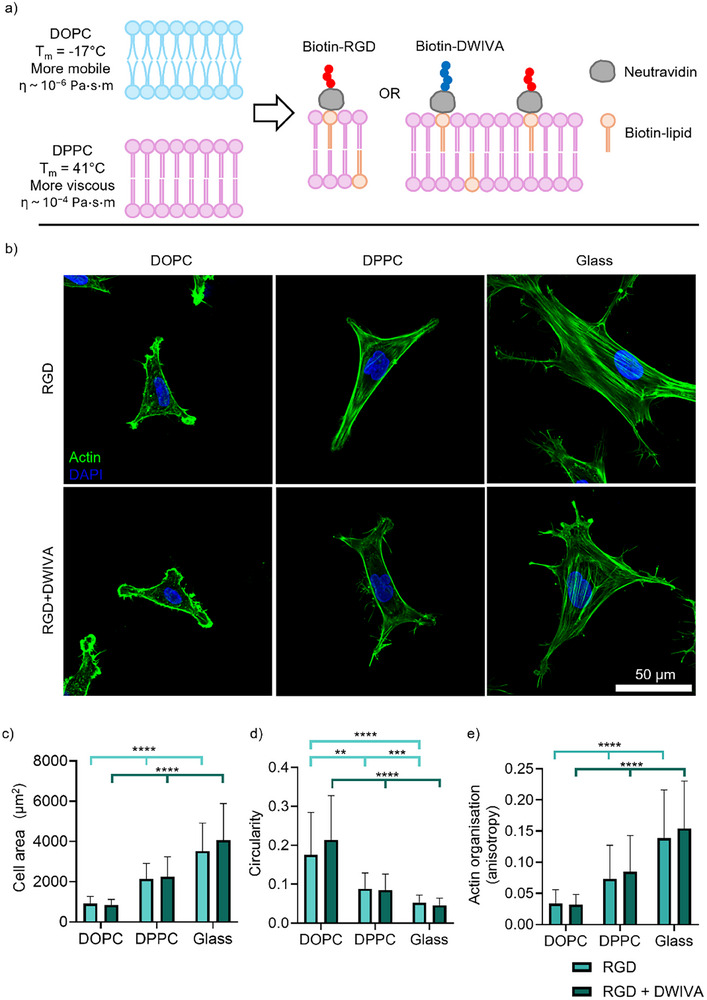
Functionalisation of supported lipid bilayers with BMP‐2‐mimetic peptide DWIVA alongside RGD allows the study of mesenchymal stem cell response to viscosity and growth factor signalling. a) Overview of the platform used with SLBs made from DOPC (mobile) or DPPC (viscous) lipids and functionalised with biotinylated DWIVA and RGD. b) Representative images of hMSCs after 24‐h culture on SLBs and non‐mobile glass, stained for actin (green) and DAPI (blue) (scale bar = 50 µm). c,d) Quantification of hMSC morphological response after 24‐h culture on SLBs and non‐mobile glass surfaces: c) Cell area (n=38, 51, 47, 49, 72, 52); d) Cell circularity (n=38, 51, 47, 49, 72, 52); and e) Organisation of actin cytoskeletal fibres (n=69, 62, 81, 73, 91, 70). Data represented as means ± SD. In graphs c,d) n represents cells; in e) n represents individual regions of the cytoskeleton, with 3‐4 measured per cell. Statistical significance was determined using D'Agostino Pearson normality test, followed by a Kruskal‐Wallis test with Dunn's multiple comparison test. ^*^ *p*<0.05, ^**^ *p*<0.01, ^***^ *p*<0.001, ^****^ *p*<0.0001.

We first evaluated cell response to SLBs functionalised with either RGD or a mixture of RGD and DWIVA (Figure 1). SLBs were prepared as previously described using either DOPC or DPPC, lipids with the same polar head group that only differ in the saturation of their hydrophobic tails, and therefore have differing transition temperatures [17]. This results in DOPC SLBs being more mobile at cell culture conditions, with an estimated viscosity of 1x10^−6^ Pa·s·m, while DPPC is more viscous (1x10^−4^ Pa·s·m) [17]. The resultant SLBs are non‐fouling surfaces that do not facilitate adsorption of proteins [36, 37], but can be functionalised with adhesive ligands and peptides via neutravidin (NA)‐biotin interactions to form homogenous 2D substrates where cells can sense differences in viscosity (Figure 1a). Glass controls, not being deformable by cells, are considered as infinitely viscous substrates.

Functionalisation of the SLBs was achieved through the addition of up to 6% mol biotinylated lipid, and the differing concentrations of biotinylated lipid did not impact SLB diffusivity and hence viscosity (Figure S1a–c), confirming previous observations [18]. The presence of adhesive ligands was demonstrated to be essential to support cell adhesion, with only a few unspread cells present in the absence of any functionalisation (Figure S2a–c). As expected, functionalisation with DWIVA alone did not support adhesion on the SLBs either (Figure S2d–f); DWIVA indeed is a GF mimetic and not an adhesive peptide. Cell adhesion was instead supported when the fibronectin‐derived adhesion sequence RGD was added. The concentration of RGD used throughout the study (2% mol) corresponds to a ligand spacing of around 12.9 nm, well within the spacing range that allows adhesion of MSCs [17, 38]. DWIVA was instead added at 4% mol: this yields a GF mimetic peptide density of around 9 pmol cm^−2^, matching levels previously indicated to be able to elicit an osteogenic effect [35]. We furthermore showed that addition of DWIVA had no effect on the density of RGD, as measured by relative fluorescent intensities of a FITC‐conjugated RGD ligand (Figure S1d–e).

It has been previously shown that changes in the viscosity or fluidity of SLBs can impact cell spreading behaviours, and MSCs respond to the higher viscosity of DPPC with higher spreading areas and stress fibre formation due to being able to exert more force upon the underlying bilayer [17, 18, 39]. Our results confirm this behaviour, with cells generally larger and more spread on DPPC than on DOPC, and even more so on glass (Figure 1b). This is confirmed by numerical analyses of cell morphology, with cell area increasing and circularity decreasing with viscosity (Figure 1c,d). Similarly, the actin cytoskeleton was found to be more organised at increasing viscosities, indicating enhanced engagement of the molecular clutch on DPPC and glass (Figure 1e). Notably, these adhesive responses were unaffected by the presence of DWIVA, as SLBs functionalised with 2% mol RGD and 4% mol DWIVA elicited the same responses as RGD‐only bilayers. Crucially, we also demonstrated SLB stability during early cell adhesion and up to 7 days; this enables the use of these dynamic models to investigate early mechanotransductive and osteogenic responses (Figure S3).

The adhesive behaviour observed for MSCs was also seen in another BMP‐2 responsive cell type, C2C12 myoblasts, with spreading morphologies responding to changes in viscosity and not to the addition of the GF mimetic (Figure S4). These results are in agreement with previous studies that have shown no effect of DWIVA on cell adhesion when the GF mimetic is randomly co‐presented with RGD, as is the case in our study, while some enhancing adhesive effects have been proposed when the ligands are presented simultaneously as a multifunctional RGD/DWIVA peptide [30, 35]. When comparing these adhesive responses to more complex viscoelastic models, the introduction of viscosity to elastic substrates has been shown to have mixed effects on cell spreading. On softer substrates, viscous cues have been shown to promote cell spreading if sufficient forces can be generated by cells to form focal adhesions or if they are able to remodel their 2D or 3D environment [40, 41]. In other studies, viscosity has been seen to hinder cell spreading, due to a dissipation of the energy exerted by cells on viscoelastic substrates [42]. DWIVA has also been introduced in viscoelastic hydrogels, but its contribution to adhesive responses has not been explored [32, 33, 43]. Taken together, the results shown here on early cellular responses show that DWIVA does not influence the adhesive and spreading behaviours of MSCs on our viscosity model.

### 2.2 DWIVA Does not Affect Viscosity‐Dependent Mechanotransduction Through Focal Adhesions or YAP

**FIGURE 2:**
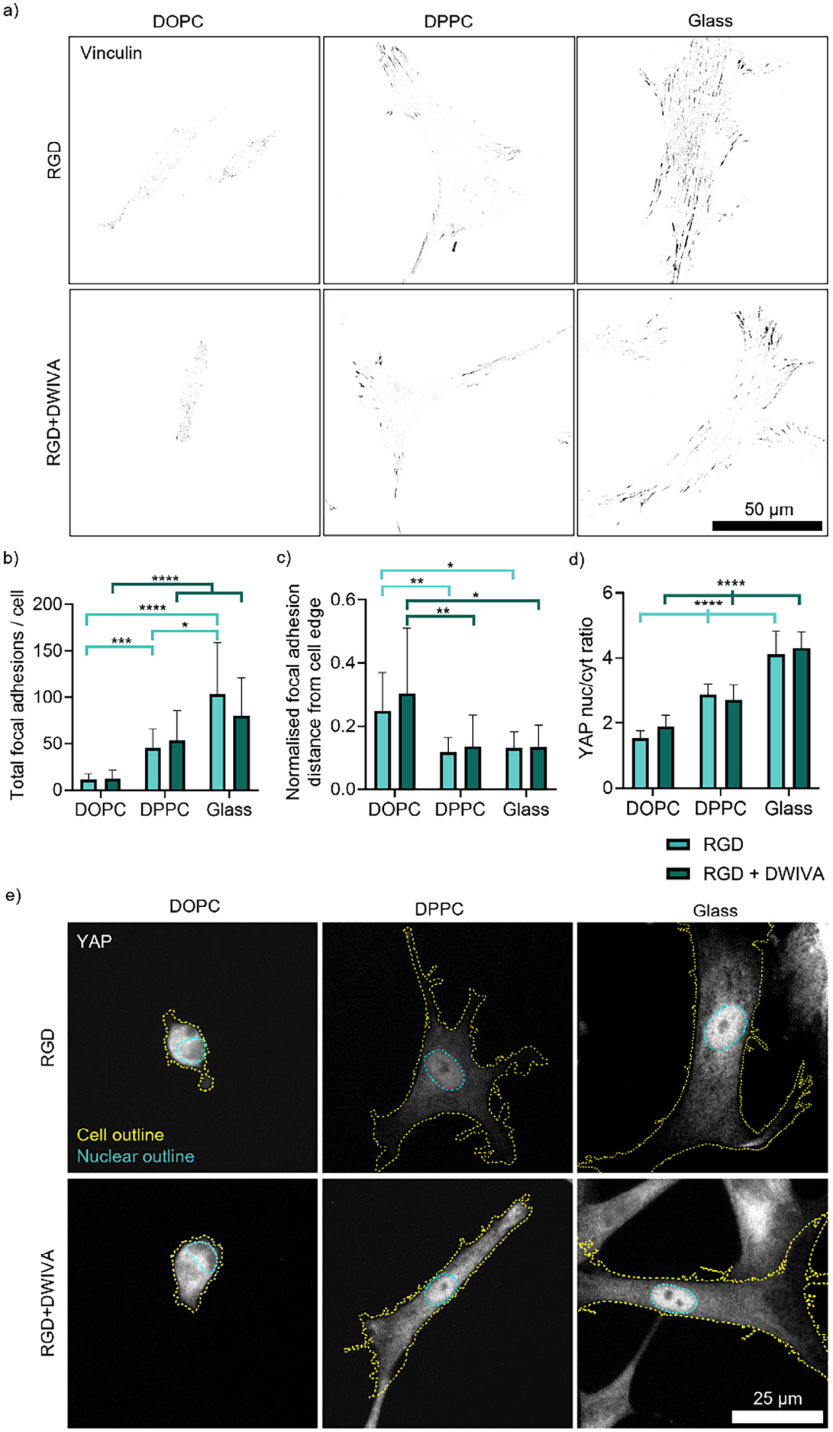
Mechanotransductive pathways are not affected by functionalisation of supported lipid bilayers with BMP‐2‐mimetic DWIVA alongside RGD. a–c) Impact of DWIVA functionalisation on focal adhesions: a) Representative images of hMSCs stained with vinculin after 24‐h culture, with a threshold and binary mask applied to visualise focal adhesions (scale bar = 50 µm); b) Quantification of total focal adhesions per cell (n = 26, 26, 26, 28, 28, 25); and c) Normalised distance of focal adhesions from the edge of the cell, where 0 is at the cell edge and 1 is at the nucleus (n = 27, 21, 21, 25, 23, 20). d,e) Impact of DWIVA functionalisation on YAP localisation: d) Quantification of YAP nuclear translocation (n=20 in all conditions); and e) Representative images of hMSCs stained for the nuclear transcription factor YAP after 24‐h culture, with cell and nuclear outlines drawn based on actin and DAPI staining, respectively (scale bar = 25 µm). Data represented as means ± SD. In all graphs, n represents individual cells. Statistical significance was determined using D'Agostino‐Pearson normality test, followed by a Kruskal‐Wallis with Dunn's multiple comparison test for b,c) and an ordinary two‐way ANOVA with Tukey's multiple comparison test for d). ^*^ *p*<0.05, ^**^ *p*<0.01, ^***^ *p*<0.001, ^****^ *p*<0.0001.

After exploring the morphological changes experienced by MSCs, we examined the effects of viscosity and functionalisation on mechanosensitive pathways, specifically FA formation and YAP localisation (Figure 2). Staining for the key FA protein vinculin indicated that FAs were larger and more numerous with increasing viscosity, while vinculin was located further away from the cell edge on DOPC (Figure 2a–c; Figure S5aS5a). Vinculin is part of the link between the actin cytoskeleton and integrins, and a stronger force applied by the cell results in a stronger and larger FA complex [9]. Hence, these results support previous observations that the molecular clutch is engaged on DPPC and glass, but not on DOPC [17, 18, 44]. Crucially, the addition of DWIVA had no effect on these trends (Figure 2a–c; Figure S5a). We further explored the dynamics of cell adhesion to the bilayers via observation of cell‐induced remodelling of fluorescently labelled NA (Figure S3a,b). On DPPC, NA reorganisation at sites of cellular adhesion was already evident after 6 h of culture (Figure S3b), leading to the formation of the mature FAs observed after 24 h. On DOPC, instead, the higher mobility of the bilayer did not support early build‐up of FAs, with evidence of local NA remodelling only at later time points (Figure S3a).

These results, together with the morphological observations of Figure 1, suggest that DWIVA has neither an inhibitory nor a synergistic effect on MSCs’ ability to adhere and engage mechanotransductive machinery on the SLBs. Indeed, growth factor‐ECM interactions that enhance cell attachment, proliferation or differentiation are not always accompanied by an effect on cell spreading or on mechanotransductive pathways [45, 46]. Our results contrast with previous literature showing that co‐presentation of RGD and DWIVA ligands results in larger and more numerous FA complexes; however, this effect was seen due to a close spatial organisation of the ligands on a non‐mobile, stiff surface [30]. Immobilised BMP‐2 was also found to enhance cell spreading and focal adhesion formation via β_3_ integrin activity; this was, however, observed on soft substrates alone [47, 48]. As the eventual common outcome of mechanotransduction is to alter gene expression within the cell, we next looked at a prominent mechanosensitive nuclear transcription factor, YAP (Figure 2d,e). This protein translocates into the nucleus in response to an increase in actin cytoskeletal tension and can affect numerous cellular outcomes, including MSC differentiation [11]. Indeed, YAP/TAZ has been seen to be involved in enabling BMP‐2 signalling via Rho‐associated protein kinase (ROCK), and a lack of YAP/TAZ nuclear translocation inhibits osteogenesis [49, 50, 51]. In our case, YAP appeared more diffuse throughout the whole cell on DOPC, while staining showed an increase in nuclear retention at increasing viscosity (Figure 2e), which was confirmed by quantifying the YAP nuclear/cytoplasmic ratio (Figure 2d). As expected, based on DWIVA having no effect on actin organisation or FA formation, there was also no difference in the nuclear translocation of YAP when surfaces were functionalised both with RGD and DWIVA compared to RGD alone (Figure 2d). This supports previous findings that, while there is overlap between YAP and growth factor signalling, translocation of YAP is independent of BMP‐2 signalling [49]. Our results therefore suggest that functionalisation of the SLBs with DWIVA has no noticeable impact on FA formation, cytoskeletal tension and on their resultant effect on YAP translocation, with surface viscosity therefore remaining the dominant factor in determining MSCs’ mechanotransductive behaviour.

### 2.3 DWIVA Elicits an Early Osteogenic Response Through Non‐Canonical BMP‐2 Signalling Only on DPPC

**FIGURE 3:**
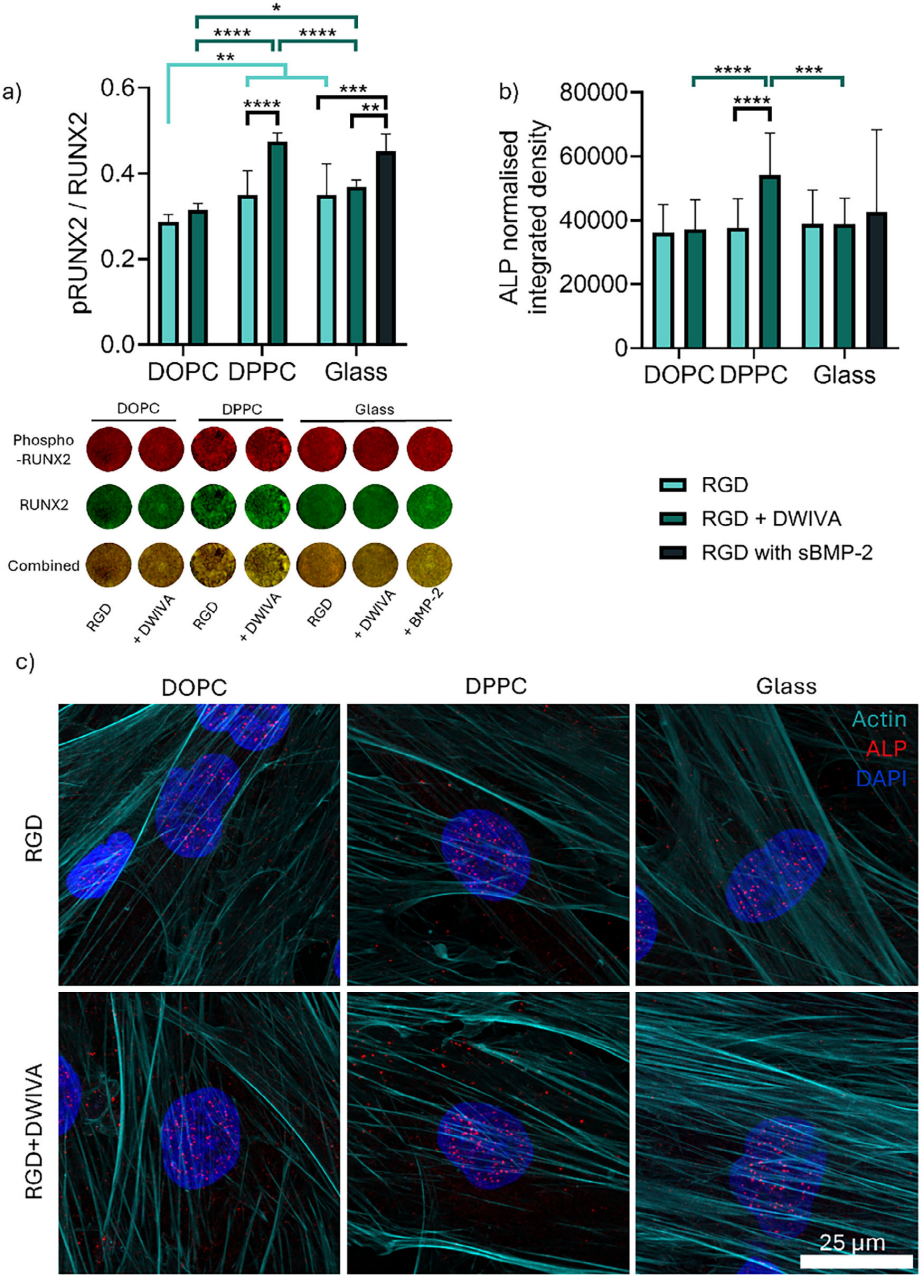
BMP‐2‐mimetic DWIVA promotes osteogenesis in hMSCs only on the viscous bilayer DPPC. a) Ratio of early osteogenic marker phosphorylated RUNX2/RUNX2 as measured by in‐cell western (ICW) after 5 days of culture (n=9 in all conditions) with representative wells for each condition. b) Integrated density values of tissue‐non‐specific ALP normalised by cell numbers after 7 days of culture (n = 15–16 in all conditions). c) Representative images of tissue‐non‐specific ALP staining (red) after 7 days of culture (scale bar = 25 µm); the actin cytoskeleton (cyan) and nuclei (blue) are also stained. Soluble BMP‐2 was added as a control on glass + RGD surfaces at a concentration of 200 ng/ml. Data represented as means ± SD. In graph a) n represents regions of interest drawn on n=3 wells; in graph b) n represents images from n=3 wells. Statistical significance was determined using D'Agostino Pearson normality test, followed by an ordinary two‐way ANOVA with Tukey's multiple comparison test, and an ordinary one‐way ANOVA with Holm‐Šidák for the glass controls. ^*^ *p*<0.05, ^**^ *p*<0.01, ^***^ *p*<0.001, ^****^ *p*<0.0001.

We evaluated the effect of DWIVA on MSC differentiation using early osteogenic markers Runt‐related transcription factor 2 (RUNX2) and alkaline phosphatase (ALP) (Figure 3); this is within the timeframe of stability of the SLBs (Figure S3) [17, 18]. RUNX2 is one of the master regulators of osteogenesis alongside osterix and can be activated within a few days of differentiation [52, 53]. Functionalisation with DWIVA elicited higher phosphorylation levels of RUNX2 after 5 days of culture on DPPC (ƞ∼1 × 10^−4^ Pa·s·m) compared to both DOPC (ƞ∼1 × 10^−6^ Pa·s·m) and glass, at levels comparable to a soluble BMP‐2 control (Figure 3a); a concentration of 200ng/ml BMP‐2 was selected for this as it was seen to activate BMP‐2 canonical signalling pathways while not impacting cell morphology (Figure S5b,c). Similarly, DWIVA elicited an increase in the levels of transcription factor osterix only on DPPC, and not on DOPC or glass (Figure S6). By day 7, ALP was upregulated on DWIVA/RGD‐functionalised DPPC surfaces compared to the rest of the samples, denoting early osteogenesis (Figure 3b,c).

We further illustrated the osteogenic potential of DWIVA using a faster differentiating cell line, C2C12 myoblasts. These cells normally default toward a myogenic lineage but will otherwise undergo osteogenesis, for example, in the presence of BMP‐2; as they differentiate faster than MSCs, they are a good model for evaluating osteogenic potential in shorter time frames [54]. After 5 days of culture, functionalisation with DWIVA resulted in a decrease in myogenic differentiation (measured as the proportion of Myosin 4‐expressing cells) only on DPPC, with no effect seen instead on DOPC or glass (Figure S7). This was accompanied by an increase in early osteogenic markers, osterix at day 3 and ALP at day 5, as well as late marker osteocalcin after 8 days of culture (Figure S8). Taken together, these results demonstrate the ability of DWIVA to elicit osteogenesis in the absence of other biochemical stimuli, while necessitating suitable physical cues [28, 30, 31]. This supports previous work showing the osteogenic effects of DWIVA, either when co‐presented as part of a bi‐mimetic ligand with RGD or incorporated into degradable 3D hydrogels [30, 33]. Critically, we also showed that this osteogenic effect requires a threshold of viscosity, with only the more viscous DPPC promoting it and not the more mobile DOPC. Limited viscosity might then explain why DWIVA seems to have no noticeable osteogenic effect in some viscoelastic hydrogels, emphasising the need for models that deconvolute the elastic and viscous contribution [32, 33, 34].

**FIGURE 4:**
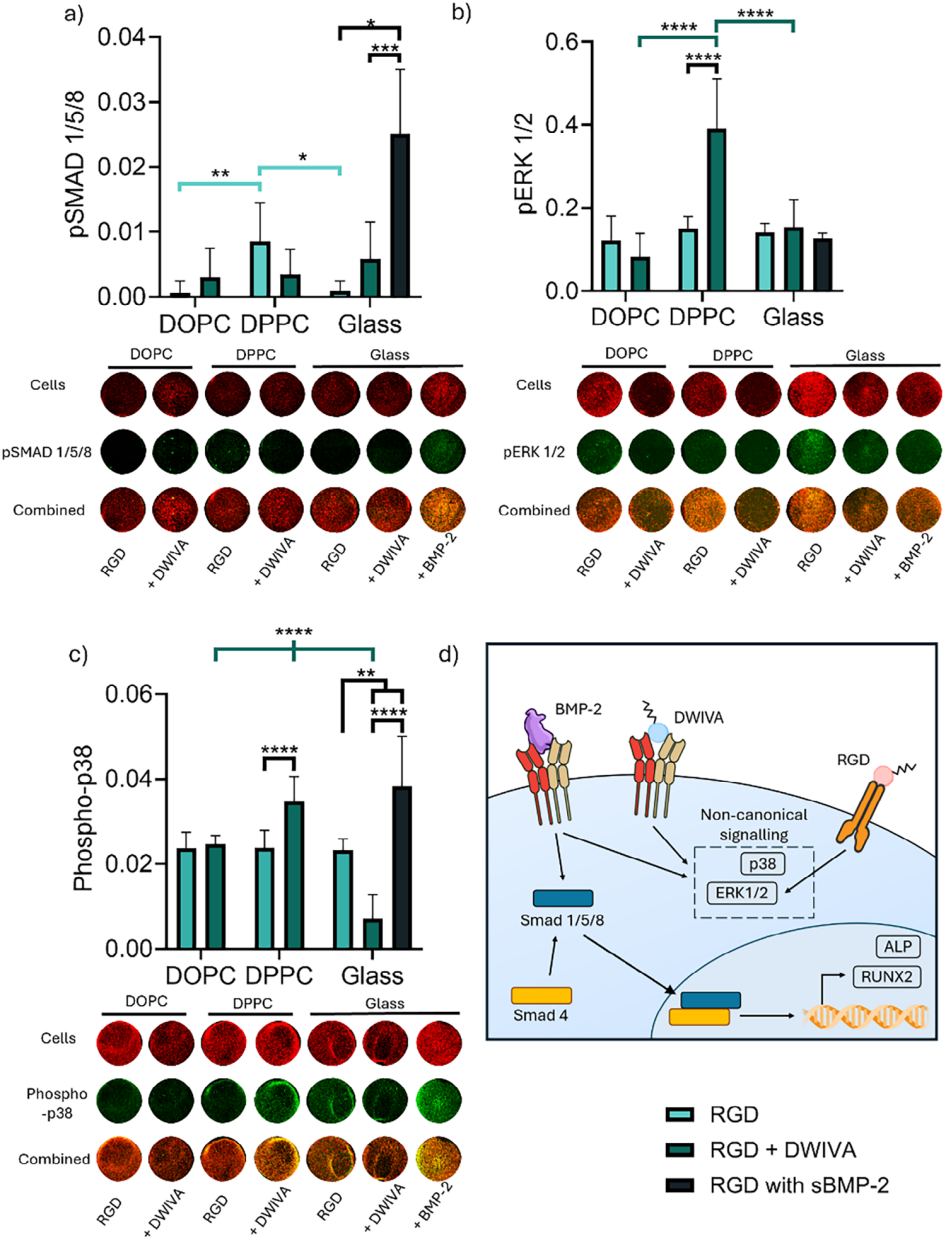
BMP‐2‐mimetic DWIVA elicits non‐canonical signalling pathways on the viscous bilayer DPPC. a) Canonical signalling protein phospho‐SMAD 1/5/8 after 90 min of culture (n=9 in all conditions) as measured by in‐cell western. b) Non‐canonical phospho‐ERK1/2 after 45 min of culture (n=9 in all conditions), and c) phospho‐p38 after 90 min of culture (n=9, 9, 9, 8, 6, 7, 9) as measured by in‐cell western. d) Schematic summarising the effect of DWIVA on BMP‐2 pathways made with public domain images obtained from NIH BIOART Source. Soluble BMP‐2 was added on glass + RGD surfaces at a concentration of 200 ng/ml. Data represented as means ± SD, and representative wells are presented for each condition. In all graphs, n represents regions of interest drawn on n=3 wells. Statistical significance was determined using D'Agostino Pearson (a,b) or a Shapiro‐Wilk (c) normality test, followed by a Kruskal‐Wallis with Dunn's multiple comparison test (a) or an ordinary two‐way ANOVA with Tukey's multiple comparison test, with an ordinary one‐way ANOVA with Holm‐Šidák for the glass controls (b,c). ^*^ *p*<0.05, ^**^ *p*<0.01, ^***^ *p*<0.001, ^****^ *p*<0.0001.

We further studied the mechanisms through which DWIVA elicits an osteogenic response on DPPC by investigating BMP‐2 signalling pathways by in‐cell western (Figure 4). Canonical BMP‐2 signalling involves direct gene transcription by Smad proteins; following BMP receptor oligomerisation, activated Smad1/5/8 form a complex with Smad4, which enters the nucleus and is involved in a wide range of functions, including upregulation of RUNX2 (Figure 4d) [55, 56]. While we observed no activation of SMAD signalling other than by the BMP‐2 control (Figure 4a), two key MAPK proteins, ERK1/2 and p38, exhibited more sustained activation after functionalisation with DWIVA on DPPC (Figure 4b,c). The lack of Smad activation in the short term seen here agrees with previous work where DWIVA was suggested to act in a Smad‐independent manner via MAP kinase p38 to induce osteogenic differentiation [35]. The MAPK pathway includes multiple proteins which can be activated in response to a range of factors, such as cellular stress and various inflammatory cytokines, as well as growth factors such as BMP‐2 [57]; in response to BMP signalling, this pathway can activate RUNX2 as well as ALP [55, 58]. It is notable that DWIVA functionalisation resulted in an increase in phosphorylated‐p38 and pERK1/2 on all surfaces at earlier time points, although this transient activation of non‐canonical signalling was lost soon after initial binding of DWIVA to BMP receptors (Figure 4b,c; Figure S9). Crucially, the increase in MAPK activation on DWIVA/RGD‐functionalised DPPC lasted longer than on either DOPC or glass. In a variety of bone precursor cell types, the activation of both p38 and ERK1/2 has been shown to be critical for ALP, and BMP‐2 stimulation results in their sustained activation; activation of p38 has been shown to peak after 1 h, while ERK1/2 exhibits fluctuating but sustained levels of activation that can last for multiple hours after simulation [59, 60, 61]. ERK1/2 can be activated in response not only to growth factor signalling but also to mechanical signals through integrins, and increased intracellular tension can result in ERK1/2‐induced activation of RUNX2 [57, 62]. Indeed, in our results, there was a significantly higher activation of ERK1/2 on glass compared to the two SLBs at the earliest time point (Figure S9d), as might be expected based on the higher levels of stress fibre formation for this condition. Taken together, these results suggest that DPPC offers insight into the conditions necessary for cells to maintain the activation of MAPK pathways via a cooperation between DWIVA‐driven signalling and physical cues, ultimately leading to osteogenic differentiation. The viscosity of DPPC in fact, allows the formation of focal adhesions and stress fibres compared to the more fluid DOPC, where the absence of sufficient cell‐generated forces ultimately results in an inability to sustain BMP‐2 signalling. Indeed, it has been previously shown that pharmacological induction of stress fibre formation in C2C12 cells enhances the activity of BMP‐2, while inhibition of stress fibre formation reduces the effect of the GF [49]. Intriguingly, our work further revealed that a static environment, such as glass, is also unable to support BMP‐2 signalling via matrix‐bound DWIVA, suggesting that the dynamic interactions provided by the SLBs are also crucial to harness its osteogenic potential.

### 2.4 Viscosity‐Driven Receptor Reorganisation Unlocks DWIVA Activity

**FIGURE 5:**
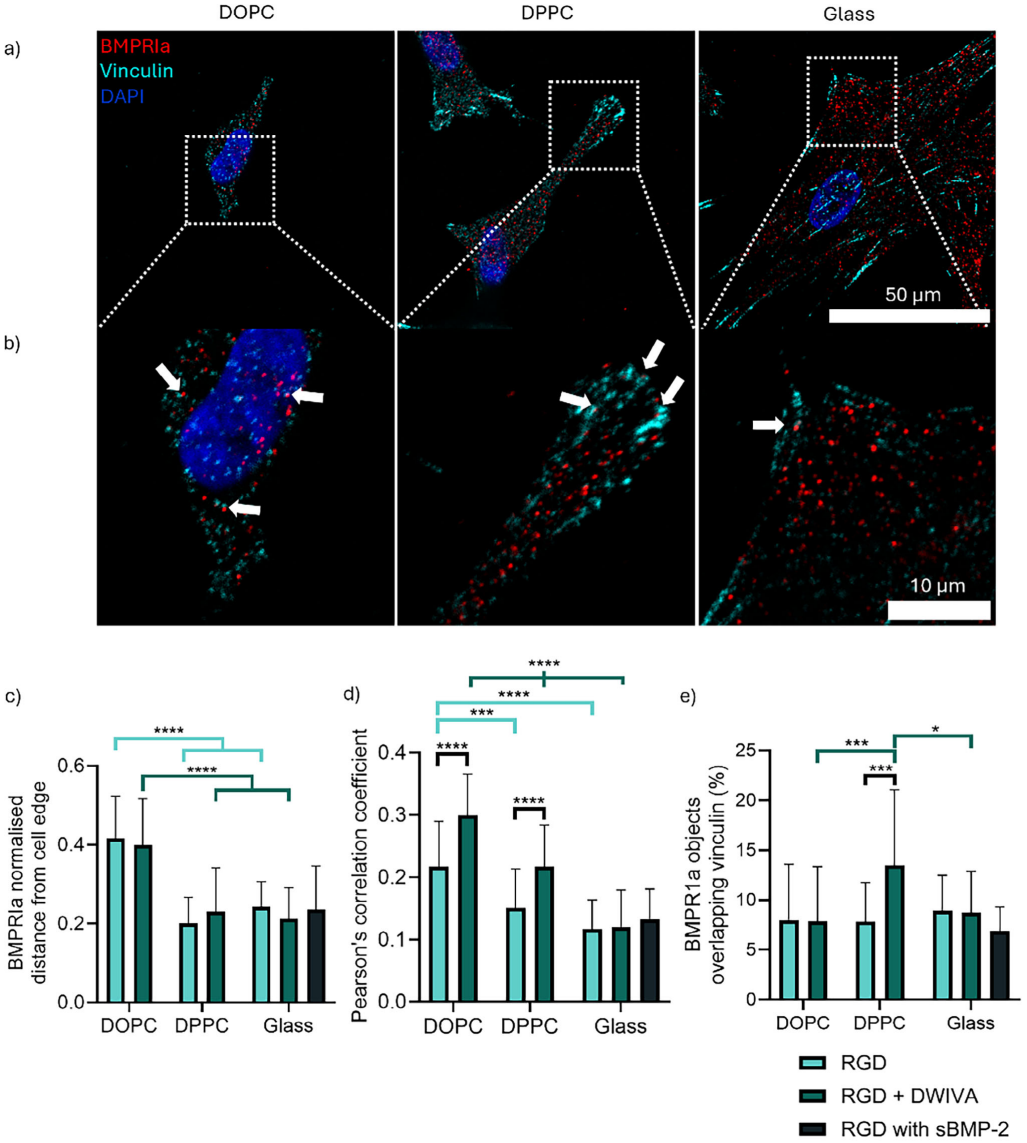
BMPRIa receptors reorganise and exhibit increased colocalisation with vinculin on supported lipid bilayers. a,b) Representative images showing hMSCs stained for BMPRIa (red), vinculin (cyan) and DAPI (blue) on surfaces with RGD+DWIVA after 24 h, including a) whole cell (scale bar = 50 µm) and b) cropped region showing cell edge (scale bar = 10 µm). White arrows indicate instances of colocalisation. c) Normalised distance of BMPRIa from the edge of the cell, where 0 is at the cell edge, and 1 is at the nucleus (n=24, 27, 19, 22, 20, 19, 17). d) Pearson's Correlation Coefficient of BMPRIa and vinculin at regions of the cell edge (n=30, 31, 30, 30, 32, 32, 31). e) Percentages of BMPRIa receptors directly overlapping with vinculin (n=28, 27, 21, 23, 23, 20, 17). Soluble BMP‐2 was added to glass + RGD surfaces at a concentration of 200 ng/ml. Data represented as means ± SD. In graphs c) and e) n represents individual cells; in graph d) n represents edge regions from individual cells. Statistical significance was determined using D'Agostino Pearson normality test, followed by an ordinary two‐way ANOVA with Tukey's multiple comparison test ^*^ *p*<0.05, ^**^ *p*<0.01, ^***^ *p*<0.001, ^****^ *p*<0.0001.

**FIGURE 6:**
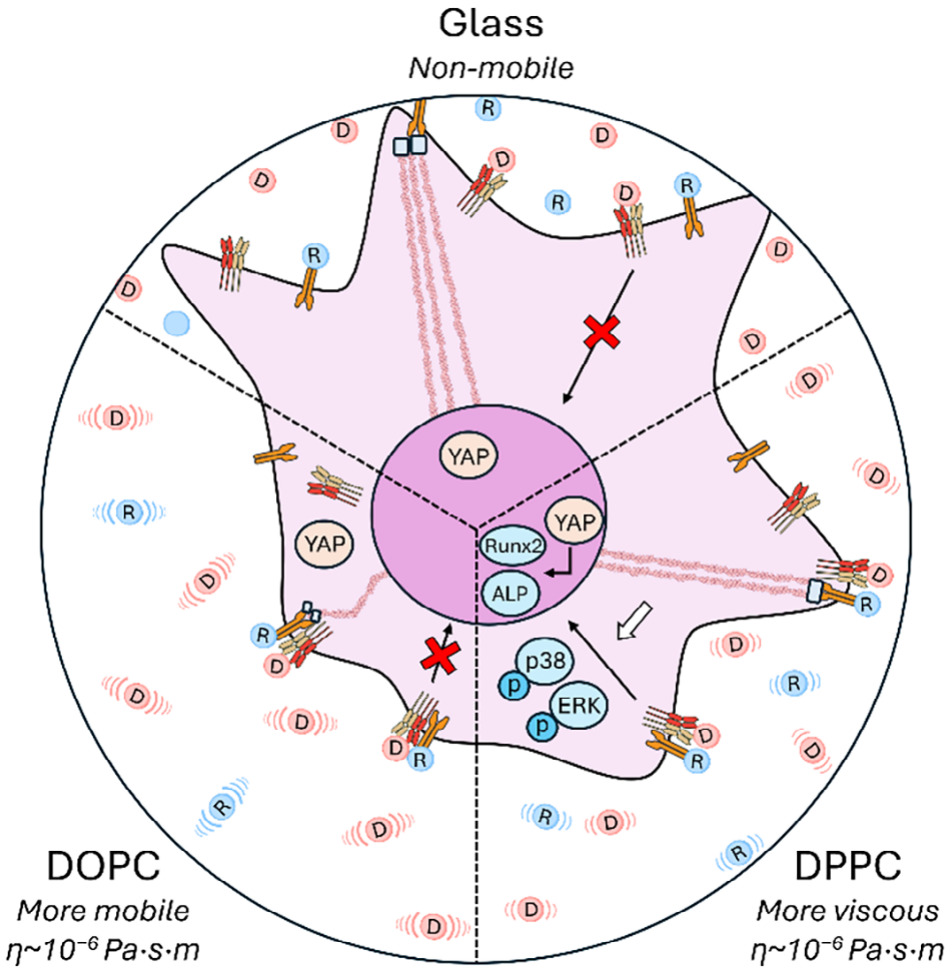
Graphical summary showing how GF and adhesive machinery interact to unlock the osteogenic effect of DWIVA on viscous DPPC and not DOPC or glass substrates. Only on DPPC is a sufficient level of cell‐generated forces accompanied by local reorganisation of ligands, allowing receptor clustering and eliciting enhanced osteogenic signalling.

To investigate whether the osteogenic effect of DWIVA seen on DPPC but not glass was related to the dynamic behaviour of the SLBs, we evaluated the localisation of BMP receptors on the surface (Figure 5). BMPRIa was selected as it is bound with high affinity by the wrist epitope of BMP‐2 from which DWIVA is derived, and plays an important role in osteogenesis through regulation of both canonical and non‐canonical BMP signalling pathways [63, 64]. Firstly, we observed that BMPRIa, similarly to vinculin (Figure 2c), was located closer to the edge of cells on DPPC and glass compared to DOPC (Figure 5a–c; Figure S10). This suggests that the presence of sufficient cell‐generated forces causes BMPRIa to translocate toward the cell edge independently of its engagement with the ligands. Furthermore, Pearson's correlation coefficient between BMPRIa and vinculin at the cell edge indicated increased colocalisation between them on both SLBs compared to glass, particularly in the presence of DWIVA on the substrate (Figure 5a–d). Crucially, the proportion of BMPRIa receptor clusters found to directly overlap with vinculin was higher on RGD/DWIVA‐functionalised DPPC compared to any of the other conditions (Figure 5e). We further investigated integrin/BMPRIa colocalisation and also found an increase in Pearson's correlation coefficient on DPPC in the presence of DWIVA (Figure S11a,b). α_v_β_3_ integrins (and not α_5_β_1_) were considered as they were found to be preferentially used by cells to bind to RGD on our substrates (Figure S11c); previous works have also observed colocalisation of BMPRI receptors with integrins at focal adhesions after BMP‐2 treatment [24, 65]. BMP receptor organisation at the cell surface has previously been shown to be dynamic, with the spatial arrangement of receptor clusters seen to influence GF signalling [66]. Further to this, BMP‐2 stimulation has been demonstrated to promote BMPRIa accumulation at focal adhesions, through a process that depends on the engagement of β_3_ integrins and which affects downstream BMP‐2 signalling pathways [67]. It has also previously been shown that modifying the lateral mobility of substrates, for example, through increasing the chain length of ‐CH_3_ modifications or the tether length of RGD bound to substrates, can enhance MSC spreading and adhesive responses via improved clustering and binding of integrins [68, 69, 70]. Our results show that the dynamic nature of the SLBs, by facilitating local reorganisation, allows these adhesive and GF machineries to co‐localise, enabling DWIVA activity through integrin‐GF crosstalk (Figure 6). While cell‐generated forces are also fundamental to support GF signalling, and indeed they promote a preferential translocation of BMPRIa receptors to the cell edge, we found that this effect alone is not sufficient to support an osteogenic response. Overall, this highlights the importance of considering ligand mobility dynamics when designing ECM mimetics, including viscoelastic hydrogels.

## 3 Conclusions

Our results demonstrate that ECM dynamics can be leveraged to harness the osteoinductive potential of the DWIVA GF‐mimetic peptide through non‐canonical MAPK BMP‐2 signalling pathways. Indeed, while adhesion‐based mechanotransductive pathways remain unaffected by matrix‐bound DWIVA presentation, osteogenic differentiation is promoted only on viscous DPPC, with no GF activity instead observed on less viscous DOPC and on non‐mobile glass. Our study indicates that a dynamic environment and a minimal threshold of cell‐generated mechanical forces are both necessary to harness DWIVA's osteogenic potential. This is achieved by altering the viscosity of the substrate using SLBs, which allow to simultaneously support the activation of mechanotransductive machinery and local reorganisation of RGD and DWIVA ligands. This enables the engagement of BMP receptors in proximity to FAs, facilitating an osteogenic response by adhering MSCs. As such, this novel viscous model has highlighted key properties of the cellular microenvironment that should be considered and optimised when designing substrates using the BMP‐2‐derived peptide DWIVA. Indeed, while native ECMs are viscoelastic, uncoupling the contribution of viscosity from elasticity has enabled a clear understanding of their role in cell response and GF activity. As such, our findings will inform future work in more complex, viscoelastic hydrogel‐based ECM models, whereby the added viscous contribution should be designed to facilitate both adequate local ligand‐receptor reorganisation and appropriate activation of mechanotransductive pathways.

## 4 Experimental Section

### 4.1 Cell Culture

Primary bone marrow‐derived hMSCs (Promocell) were cultured up to passage 5 in DMEM with high glucose (Gibco), 1% L‐glutamine (2 mm; Gibco), 0.88% Penicillin/Streptomycin (Pen/Strep; 88 units/ml Penicillin, 88 µg/ml Streptomycin; Gibco), Amphotericin B (278 ng/ml; Gibco), sodium pyruvate (1 mm; Gibco), 1% non‐essential amino acids (100 µm per amino acid; Gibco) and 10% foetal bovine serum (FBS) (Gibco). Cells were serum‐starved one day prior to experiments. Cells were seeded at 10 000/cm^2^ for most experiments or 50 000/cm^2^ for use in in‐cell westerns in FBS‐free culture media; 10% FBS was added to the media for cultures longer than 24 h. Soluble BMP‐2 (Bio‐Techne) was added at a concentration of 200 ng/ml for differentiation and signalling experiments. C2C12 myoblast cells were cultured up to passage 30 in DMEM with high glucose (with 1% Pen/Strep, 1% L‐glutamine, 20% FBS). For C2C12 differentiation experiments, cells were seeded at a density of 20 000/cm^2^ in media with 1% FBS, with 200 ng/ml BMP‐2 or 1% ITS‐X (Gibco) added as an osteogenic or myogenic control, respectively.

### 4.2 Liposome Production

Solutions of lipid vesicles were created containing either DOPC (1,2‐dioleoyl‐sn‐glycero‐3‐phosphocholine) or DPPC (1,2‐dipalmitoyl‐sn‐glycero‐3‐phosphocholine) (Avanti Polar Lipids). For each lipid mixture, 2 mg of the respective lipid was mixed with a biotinylated lipid – 16:0 Biotinyl Cap PE (Avanti Polar Lipids) – at different molar concentrations to allow functionalisation of ligands (2% for RGD, 4% for DWIVA, 6% for both RGD+DWIVA, Table S1). The biotinylated lipid was stored at a concentration of 5.5 mg/ml in a solution of CHCl_3_:CH_3_OH: H_2_O (65:35:8), from which a more diluted stock containing lipid:CHCl_3_:CH_3_OH at a ratio of 7:4:69 was made for more regular use. A fluorescent lipid – TopFluor PE (Avanti Polar Lipids) – was added at a molar concentration of 0.1% to measure diffusion and stability of the bilayers. Lipid cakes were formed by drying the solutions under N_2_ gas and further drying in a vacuum for >1 h at room temperature (RT). Lipid cakes were resuspended in 750 µl rehydration buffer (10 mm Tris + 150 mm NaCl in MilliQ water adjusted to a pH of 7.4) at RT for DOPC and 70°C for DPPC. This produced a mixture of liposomes of varying sizes, while small unilamellar vesicles of less than 90nm are needed to form bilayers [71]. The mixtures were therefore extruded through polycarbonate membranes (Whatman Nucleopore Track‐etched membrane, Avanti Polar Lipids) – 100 nm followed by 50 nm for DPPC, and 50 nm for DOPC – a minimum of 11 times and stored in the fridge until use for up to 2 weeks.

### 4.3 Surface Preparation

SLBs were formed in wells on RCA‐cleaned 30mm coverslips; wells were formed by binding polydimethylsiloxane (PDMS) shapes to the coverslips. PDMS was formed from a 10:1 ratio of PDMS elastomer and crosslinker (Sylgard 184, Farnell) mixed in a large petri dish, degassed under vacuum for 30 min and cured at 70°C for >1 h. 27 mm discs were cut out using a metal stamp and three 9 mm diameter wells were cut into each of these to create the wells that would house the SLBs. The resulting shapes (Figure S12) were cleaned in ethanol, dried, and bound to the coverslips following 20 s surface activation using a handheld plasma corona (BD‐20V, Electro‐Technic Products).

### 4.4 Bilayer Formation

SLBs were formed through the vesicle deposition method. PDMS/glass surfaces in a 6‐well plate underwent surface activation in an oxygen asher (HPT‐200 Oxygen Plasma Treatment System, Henniker Plasma) set at 80 W power and 50 sccm flow for 10 min. These were then UV sterilised, and SLBs were formed inside a sterile hood. PBS, fusion (F)‐buffer (10 mm Tris + 300 mm NaCl + 10 mm MgCl_2_ in MilliQ water adjusted to a pH of 7.4) and DPPC liposomes were kept at 70°C until use. Liposomes were diluted in sterile F‐buffer (1 in 30 for DOPC, 1 in 15 for DPPC) and passed through a 200 nm filter for sterilisation. PDMS wells were then filled with liposome mixtures and kept at 70°C for 25 min before washing with warm F‐buffer (×7) and warm PBS (×5) in preparation for surface functionalisation.

### 4.5 Non‐Mobile Glass Control

A gaseous silanisation protocol was followed to prepare the non‐mobile glass controls for functionalisation. Biotin‐polyethene glycol (PEG)‐silane (Nanocs) was dissolved in absolute ethanol at a concentration of 10 mg/ml. PDMS/glass surfaces were placed in a silane desiccator alongside a square glass coverslip containing 0.5 mg of silane solution per sample. A vacuum was formed, and samples were left in the sealed desiccator for >2 h. Silanised samples were UV sterilised and washed with PBS just prior to surface functionalisation.

### 4.6 Surface Functionalisation

Functionalisation occurred by exploiting avidin‐biotin interactions, with the addition of neutravidin (NA, Fisher Scientific) to interact with the biotinylated lipid (SLBs) or biotinylated silane (glass controls) of the surfaces. 5 mg/ml neutravidin was diluted 1 in 50 with PBS for SLBs and 1 in 10 for glass controls. For visualisation of the NA layer, Texas Red NA (Invitrogen) was added at a concentration of 0.02 mg/ml to both SLBs and glass. After adding to each well, plates were left for 30 min at RT followed by PBS washes (×3). Biotinylated peptides (Genscript, Table 1) were diluted to a concentration of 1.92 µg/ml in PBS, and 50 µl was added to SLB wells, while a 1:2 RGD: DWIVA molar ratio was used for functionalisation with both peptides. For glass, the percentage of functionalisation was controlled through the addition of biotin‐PEG to peptide mixes at ratios of 98:2 (biotin‐PEG:biotin‐RGD), 96:4 (biotin‐PEG:biotin‐DWIVA) or 94:2:4 (biotin‐PEG:biotin‐RGD:biotin‐DWIVA). For visualisation of RGD distribution, RGD was replaced with a FITC‐RGD ligand at the same molar ratios as above. After leaving for 30 min at RT, plates were washed with PBS (×2) and serum‐free media and warmed to 37°C ready for the addition of cells.

**TABLE 1:** Genscript peptide sequences used for functionalisation of SLBs.

Peptide	Sequence	Mw (g/mol)
*Biotin‐PEG*	*biotin{PEG2}*	*389.47*
*Biotin‐DWIVA*	*biotin{PEG2}DWIVA*	*974.17*
*Biotin‐RGD*	*biotin{PEG2}GCGYGRGDSPG*	*1396.53*
*Biotin RGD‐FITC*	*biotin{PEG2}GCGYGRGDSPG{PEG2}{Lys(FITC)*	*2059.15*

### 4.7 Immunofluorescence

Cells were fixed with 4% paraformaldehyde (20 mins), permeabilised with 0.1% Triton X‐100 (Merck) (10 mins), and blocked with 1% BSA (30 mins). Wells were washed with PBS (×3) in between each step. Primary antibodies used were, anti‐vinculin (1:400, mouse monoclonal; Sigma–Aldrich V9131), anti‐YAP (1:100, mouse monoclonal; Santa Cruz Biotechnology sc‐101199), anti‐ALPL (1:50, rabbit polyclonal; Proteintech 11187‐1‐AP), anti‐osterix (1:100, mouse monoclonal; Santa Cruz Biotechnology sc‐393325), anti‐Myosin 4 (1:100, mouse monoclonal; Invitrogen 14‐6503‐82), anti‐osteocalcin (1:200, rabbit polyclonal; Proteintech 23418‐1‐AP), anti‐BMPRIa (1:50, rabbit polyclonal; Invitrogen 38‐6000), anti‐integrin α_v_β_3_ (1:200, mouse monoclonal; Biotechne MAB3050), anti‐integrin α_5_β_1_ (1:50, rat monoclonal; Sigma–Aldrich MAB2514). Secondary antibodies used were Cy3 anti‐mouse (1:100, rabbit polyclonal; Jackson ImmunoResearch 315‐165‐003), Cy3 anti‐rabbit (1:100, goat polyclonal; Jackson ImmunoResearch 111‐165‐003), Alexa Fluor 488 anti‐mouse (1:200, goat polyclonal; Invitrogen A11001), Alexa Fluor 488 anti‐rabbit (1:200, donkey anti‐rabbit; Invitrogen A32790), Alexa Fluor 488 anti‐rat (1:250, chicken polyclonal; Invitrogen A21470), and Alexa Fluor 488 phalloidin (1:100; Fisher scientific A12379). Antibodies were added for 1 h on a shaker before 5 washes (spaced 5 mins apart) with PBS. DAPI‐containing mounting media (Vectashield) was added to each well for visualisation of cell nuclei. A Zeiss Axio Observer Z1 microscope (10x, 20x, 40x lenses) was used for imaging of cell morphology, YAP nuclear translocation and differentiation markers Myosin 4 and ALP. A Zeiss LSM 980 in confocal mode was used to image the surface of the bilayers (20x, 40x oil and 63x oil objectives) and for imaging of vinculin, receptor staining and representative images, while the widefield mode was used for imaging of differentiation marker osterix.

### 4.8 In‐Cell Western

Cells were fixed with 4% paraformaldehyde (15 mins at 37°C), permeabilised with an in‐cell western permeabilisation buffer (10.3 g sucrose, 0.292 g NaCl, 0.006 g MgCl_2_, 0.476 g Hepes in 100ml ml PBS, adjusted to pH 7.2, with 0.5 ml Triton X‐100 added last) (10 mins), and blocked with 1% milk protein (Milfresh) (90 mins). Wells were washed with PBS (×3) in between each step. Primary antibodies used were anti‐RUNX2 (1:100, mouse monoclonal; Santa Cruz Biotechnology sc‐390351), anti‐phospho‐RUNX2 (1:250, rabbit polyclonal; Invitrogen PA5‐105642), anti‐phospho SMAD1/5/9 (1:800, rabbit monoclonal; Cell Signaling #13820), anti‐phospho‐ERK1/2 (1:200, rabbit monoclonal; Cell Signaling #4370), anti‐phospho‐p38 (1:800, rabbit monoclonal; Cell Signaling #4511), and were added overnight at 4°C. Secondary antibodies used were IRDye 800CW Goat anti‐rabbit (1:800, Li‐cor 926‐32211), IRDye 800CW Donkey anti‐mouse (1:800, Li‐cor 926‐32212), IRDye 680RD Donkey anti‐mouse (1:800; Li‐cor 926‐68072), CellTag 700 Stain (1:500; Li‐cor 926‐41090), and were left for 1 h on a shaker, with PBS washes (5×, spaced 5 mins apart) after each. Samples were then dried and imaged with a Li‐Cor Odyssey M and Image Studio Lite 5.2 used for analysis. Three circles were drawn in each of the wells (for a total of 9 circles per condition), and the resulting fluorescence values from 700 and 800 channels were saved. An average background fluorescence value for each channel was obtained from cell‐free wells and subtracted from the total fluorescence for each condition.

### 4.9 Cell Counts and Morphology

After setting a binary mask on 10x nuclear images using Fiji, the ‘analyse particles’ tool was used to count the number of cells present, with the size set as 50‐infinity µm^2^. For cell morphology, a threshold was set on the 20x actin channel, with the ‘tracing’ and ‘measure’ tools used to select and measure area and shape descriptors of individual cells. Actin fibre organisation was analysed by measuring the anisotropy of 3‐4 polygonal shapes within the cytoskeleton of each cell using the FibrilTool plugin for Fiji (Figure S13) [72].

### 4.10 Focal Adhesions

A cell outline was obtained from actin images and saved to the ROI manager using Fiji. Vinculin channels were made 8‐bit, had their background subtracted (rolling ball radius = 20), and the image was converted to a binary mask using the same threshold for every condition. ‘Analyse particles’ (with the ‘fit ellipse’ measurement set) was used to obtain data on all the particles within the cell area, and only those with a major axis of at least 1 µm were considered as focal adhesions [73].

### 4.11 YAP Nuclear Translocation

A cell outline was obtained from actin images, and integrated density values for the whole cell were measured on the YAP channel after subtracting background (rolling ball radius = 50) using Fiji. Integrated density values for the nucleus were obtained after obtaining a nuclear outline from the DAPI channel. The following formula was used to generate a ratio of nuclear/cytoplasmic YAP:

$$\begin{array}{ccc} & & \frac{YAP^{nuc}\;integrated\;density}{nuclear\;area}/\\ & & \frac{YAP^{cell}\;integrated\;density-YAP^{nuc}\;integrated\;density}{\left(Whole\;cell\;area-nuclear\;area\right)} \end{array}$$

### 4.12 Quantification of Staining Intensity

Cell outlines were obtained from actin images, and mean grey values (total integrated density divided by the area of the cell) were obtained for whole cells on the channel of interest after subtracting background (rolling ball radius = 50) using Fiji.

### 4.13 Distances of BMPRIa/Vinculin to Cell Edge

Multichannel images stained for BMPRIa, vinculin and DAPI were analysed with the use of a Fiji macro, ‘Shortest distance to edge and nucleus’, adapted from [74]. In short, cell and nuclear outlines were obtained for each cell. All of the BMPRIa or vinculin objects within a cell were added to a multipoint ROI, and then the shortest distance to both the cell outline and nuclear outline for each object was measured. Normalised distances from the cell edge represent the distance to the cell edge as a proportion of the total distance to both the edge and nucleus, with 0 being next to the cell edge and 1 being at the nucleus. A more complete description of how the analysis was performed is available in the Supporting Information.

### 4.14 BMPRIa/Vinculin Colocalisation

Multichannel images stained for BMPRIa, vinculin and DAPI were analysed with the use of a Fiji macro, ‘Shortest distance to other objects’, adapted from [74]. In short, cell outlines were obtained from each cell. BMPRIa and vinculin objects within each cell were obtained after generating a binary mask on the corresponding channel. The distance of each BMPRIa object to the nearest vinculin object was measured, with a distance of 0 µm corresponding to BMPRIa objects found to be overlapping with vinculin. A more complete description of how the analysis was performed is available in the Supporting Information.

### 4.15 Pearson's Correlation Coefficient (PCC)

Regions at the cell edge were drawn on multichannel images using the polygon selections tool in Fiji, with 1‐2 regions drawn per cell and added to the ROI manager. The ‘subtract background’ function (rolling ball radius = 50) was applied to both channels of interest and the ‘Image Correlator’ plugin [75] was used to give PCC values for the two images (BMPRIa and vinculin or BMPRIa and α_v_β_3_ integrins).

### 4.16 Statistical Analysis

GraphPad Prism 10 was used to generate graphs (presented as mean ± standard deviation) and perform all statistical analyses. If data were seen to be normally distributed using a D'Agostino‐Pearson omnibus test, a two‐way ANOVA with Tukey's was used alongside an ordinary one‐way ANOVA with Holm‐Sidak on glass to compare the BMP‐2 controls. Otherwise, data were assessed using a one‐way ANOVA with Kruskal‐Wallis.

## Conflicts of Interest

The authors declare no conflict of interest.

## Supporting information




**Supporting File**: smll72188‐sup‐0001‐SuppMat.docx

## Data Availability

The data that support the findings of this study are available in Enlighten Research Data at https://doi.org/10.5525/gla.researchdata.2127.

## References

[1] L. Li , S. Wang , Y. Chen , et al., “Hydrogels Mimicking the Viscoelasticity of Extracellular Matrix for Regenerative Medicine: Design, Application, and Molecular Mechanism,” Chemical Engineering Journal 498 (2024): 155206.

[2] Y. Zhao , Q. Wu , C. Zhao , H. Zhou , and L. Wu , “Progress of Structural Scaffold Biomaterials for Bone Tissue Defect Repair: A Cutting‐Edge Review,” Composite Structures 349‐350 (2024): 118542.

[3] A. J. Engler , S. Sen , H. L. Sweeney , and D. E. Discher , “Matrix Elasticity Directs Stem Cell Lineage Specification,” Cell 126 (2006): 677–689. 16923388 10.1016/j.cell.2006.06.044

[4] O. Chaudhuri , J. Cooper‐White , P. A. Janmey , D. J. Mooney , and V. B. Shenoy , “Effects of Extracellular Matrix Viscoelasticity on Cellular Behaviour,” Nature 584 (2020): 535–546. 32848221 10.1038/s41586-020-2612-2 PMC7676152

[5] K. Bera , A. Kiepas , I. Godet , et al., “Extracellular Fluid viscosity Enhances Cell Migration and Cancer Dissemination,” Nature 611 (2022): 365–373. 36323783 10.1038/s41586-022-05394-6 PMC9646524

[6] F. Schulze , A. Lang , J. Schoon , G. I. Wassilew , and J. Reichert , “Scaffold Guided Bone Regeneration for the Treatment of Large Segmental Defects in Long Bones,” Biomedicines 11 (2023): 325. 36830862 10.3390/biomedicines11020325 PMC9953456

[7] S. Seetharaman and S. Etienne‐Manneville , “Integrin Diversity Brings Specificity in Mechanotransduction,” Biology of the Cell 110 (2018): 49–64. 29388220 10.1111/boc.201700060

[8] T. Mitchison and M. Kirschner , “Cytoskeletal Dynamics and Nerve Growth,” Neuron 1, (1988): 761–772. 3078414 10.1016/0896-6273(88)90124-9

[9] D. L. Huang , N. A. Bax , C. D. Buckley , W. I. Weis , and A. R. Dunn , “Vinculin Forms A Directionally Asymmetric Catch Bond with F‐Actin,” Science 357 (2017): 703–706. 28818948 10.1126/science.aan2556 PMC5821505

[10] Z. Sun , S. S. Guo , and R. Fässler , “Integrin‐Mediated Mechanotransduction,” Journal of Cell Biology 215 (2016): 445–456. 27872252 10.1083/jcb.201609037 PMC5119943

[11] S. Dupont , L. Morsut , M. Aragona , et al., “Role of YAP/TAZ in Mechanotransduction,” Nature 474 (2011): 179–183. 21654799 10.1038/nature10137

[12] M. Cantini , H. Donnelly , M. J. Dalby , and M. Salmeron‐Sanchez , “The Plot Thickens: The Emerging Role of Matrix Viscosity in Cell Mechanotransduction,” Advanced Healthcare Materials 9 (2020): 1901259. 10.1002/adhm.201901259 31815372

[13] O. Chaudhuri , L. Gu , D. Klumpers , et al., “Hydrogels with Tunable Stress Relaxation Regulate Stem Cell Fate and Activity,” Nature Materials 15 (2016): 326–334. 26618884 10.1038/nmat4489 PMC4767627

[14] J. Whitehead , K. H. Griffin , M. Gionet‐Gonzales , C. E. Vorwald , S. E. Cinque , and J. K. Leach , “Hydrogel Mechanics are a Key Driver of Bone Formation by Mesenchymal Stromal Cell Spheroids,” Biomaterials 269 (2021): 120607. 33385687 10.1016/j.biomaterials.2020.120607 PMC7870573

[15] G. Koçer and P. Jonkheijm , “Guiding hMSC Adhesion and Differentiation on Supported Lipid Bilayers,” Adv Healthc Mater 6 (2017): 1600862. 10.1002/adhm.201600862 27893196

[16] S. H. Kao , S. Y. Liang , P. L. Cheng , and H. L. Tu , “Surface Viscosity‐Dependent Neurite Initiation in Cortical Neurons,” Advanced Biology 6 (2022): 2101325. 10.1002/adbi.202101325 35362269

[17] M. Bennett , M. Cantini , J. Reboud , J. M. Cooper , P. Roca‐Cusachs , and M. Salmeron‐Sanchez , “Molecular Clutch Drives Cell Response to Surface Viscosity,” Proceedings of the National Academy of Sciences 115 (2018): 1192–1197. 10.1073/pnas.1710653115 PMC5819391 29358406

[18] E. Barcelona‐Estaje , M. A. G. Oliva , F. Cunniffe , et al., “N‐Cadherin Crosstalk with Integrin Weakens the Molecular Clutch in Response to Surface Viscosity,” Nature Communications 15 (2024): 1–12. 10.1038/s41467-024-53107-6 PMC11479646 39394209

[19] A. Cipitria and M. Salmeron‐Sanchez , “Mechanotransduction and Growth Factor Signalling to Engineer Cellular Microenvironments,” Advanced Healthcare Materials 6 (2017): 1700052. 10.1002/adhm.201700052 28792683

[20] É. R. Oliveira , L. Nie , D. Podstawczyk , et al., “Advances in Growth Factor Delivery for Bone Tissue Engineering,” International Journal of Molecular Sciences 22 (2021): 903. 33477502 10.3390/ijms22020903 PMC7831065

[21] A. W. James , G. LaChaud , J. Shen , et al., “A Review of the Clinical Side Effects of Bone Morphogenetic Protein‐2,” Tissue Engineering Part B: Reviews 22 (2016): 284–297. 26857241 10.1089/ten.teb.2015.0357 PMC4964756

[22] D. Halloran , H. W. Durbano , and A. Nohe , “Bone Morphogenetic Protein‐2 in Development and Bone Homeostasis,” Journal of Developmental Biology 8 (2020 ): 19. 32933207 10.3390/jdb8030019 PMC7557435

[23] K. Kundu , S. V. Jaswandkar , D. R. Katti , and K. S. Katti , “Initial Upsurge of BMPs Enhances Long‐Term Osteogenesis in In‐Vitro Bone Regeneration,” Materialia 26 (2022): 101576.

[24] V. Llopis‐Hernández , M. Cantini , C. González‐García , et al., “Material‐Driven Fibronectin Assembly for High‐Efficiency Presentation of Growth Factors,” Science Advances 2 (2016): 1600188. 10.1126/sciadv.1600188 PMC5001810 27574702

[25] T. Crouzier , L. Fourel , T. Boudou , C. Albigès‐Rizo , and C. Picart , “Presentation of BMP‐2 From a Soft Biopolymeric Film Unveils its Activity on Cell Adhesion and Migration,” Advanced Materials 23 (2011): H111–H118. 21433098 10.1002/adma.201004637

[26] O. F. Zouani , J. Kalisky , E. Ibarboure , and M. C. Durrieu , “Effect of BMP‐2 From Matrices of Different Stiffnesses for the Modulation of Stem Cell Fate,” Biomaterials 34 (2013): 2157–2166. 23290467 10.1016/j.biomaterials.2012.12.007

[27] E. Brauer , A. Herrera , R. Fritsche‐Guenther , et al., “Mechanical Heterogeneity in a Soft Biomaterial Niche Controls BMP2 Signaling,” Biomaterials 309 (2024): 122614. 38788455 10.1016/j.biomaterials.2024.122614

[28] K. B. Seims , N. K. Hunt , and L. W. Chow , “Strategies to Control or Mimic Growth Factor Activity for Bone, Cartilage, and Osteochondral Tissue Engineering,” Bioconjugate Chemistry 32 (2021): 861–878. 33856777 10.1021/acs.bioconjchem.1c00090

[29] J.‐Y. Lee , J.‐E. Choo , Y.‐S. Choi , et al., “Osteoblastic Differentiation of Human Bone Marrow Stromal Cells in Self‐Assembled BMP‐2 Receptor‐Binding Peptide‐Amphiphiles,” Biomaterials 30 (2009): 3532–3541. 19345406 10.1016/j.biomaterials.2009.03.018

[30] L. Oliver‐Cervelló , H. Martin‐Gómez , N. Mandakhbayar , et al., “Mimicking Bone Extracellular Matrix: From BMP‐2‐Derived Sequences to Osteogenic‐Multifunctional Coatings,” Advanced Healthcare Materials 11 (2022): 2201339. 35941083 10.1002/adhm.202201339 PMC11468143

[31] K. A. Gultian , R. Gandhi , K. DeCesari , et al., “Injectable Hydrogel with Immobilized BMP‐2 Mimetic Peptide for Local Bone Regeneration,” Frontiers in Biomaterials Science 1 (2022): 948493. 37090104 10.3389/fbiom.2022.948493 PMC10120851

[32] J. M. Lowen , E. E. Wheeler , N. K. Shimamoto , et al., “Functionalized Annealed Microgels for Spatial Control of Osteogenic and Chondrogenic Differentiation,” Advanced Functional Materials 34 (2024): 2311017. 40800235 10.1002/adfm.202311017 PMC12341486

[33] S. Love , K. Gultian , U. Jalloh , A. Stevens , T. W. B. Kim , and S. L. Vega , “Mesenchymal Stem Cells Enhance Targeted Bone Growth From Injectable Hydrogels with BMP‐2 Peptides,” Journal of Orthopaedic Research 42 (2024): 1599–1607. 38323639 10.1002/jor.25798 PMC11161325

[34] C. M. Madl , M. Mehta , G. N. Duda , S. C. Heilshorn , and D. J. Mooney , “Presentation of BMP‐2 Mimicking Peptides in 3D Hydrogels Directs Cell Fate Commitment in Osteoblasts and Mesenchymal Stem Cells,” Biomacromolecules 15 (2014): 445–455. 24400664 10.1021/bm401726u PMC3930060

[35] L. Oliver‐Cervelló , H. Martin‐Gómez , L. Reyes , et al., “An Engineered Biomimetic Peptide Regulates Cell Behavior by Synergistic Integrin and Growth Factor Signaling,” Advanced Healthcare Materials 10 (2021): 2001757. 10.1002/adhm.202001757 33336559

[36] S. H. Kim and E. I. Franses , “New Protocols for Preparing Dipalmitoylphosphatidylcholine Dispersions and Controlling Surface Tension and Competitive Adsorption with Albumin at the Air/Aqueous Interface,” Colloids and Surfaces B: Biointerfaces 43 (2005): 256–266. 15979858 10.1016/j.colsurfb.2005.05.006

[37] A. Van Der Heiden , G. M. Willems , T. Lindhout , A. P. Pijpers , and L. H. Koole , “Adsorption of Proteins Onto poly(Ether Urethane) with a Phosphorylcholine Moiety and Influence of Preadsorbed Phospholipid,” Journal of Biomedical Materials Research 40 (1998): 195–203. 9549614 10.1002/(sici)1097-4636(199805)40:2<195::aid-jbm4>3.0.co;2-g

[38] E. Cavalcanti‐Adam , T. Volberg , A. Micoulet , H. Kessler , B. Geiger , and J. P. Spatz , “Cell Spreading and Focal Adhesion Dynamics Are Regulated by Spacing of Integrin Ligands,” Biophysical Journal 92 (2007): 2964–2974. 17277192 10.1529/biophysj.106.089730 PMC1831685

[39] W. Hao , J. Han , Y. Chu , et al., “Lower Fluidity of Supported Lipid Bilayers Promotes Neuronal Differentiation of Neural Stem Cells by Enhancing Focal Adhesion Formation,” Biomaterials 161 (2018): 106–116. 29421547 10.1016/j.biomaterials.2018.01.034

[40] Z. E. Gong , S. E. Szczesny , S. R. Caliari , et al., “Matching Material and Cellular Timescales Maximizes Cell Spreading on Viscoelastic Substrates,” Proceedings of the National Academy of Sciences 115 (2018): E2686–E2695. 10.1073/pnas.1716620115 PMC5866566 29507238

[41] A. N. Borelli , M. W. Young , B. E. Kirkpatrick , et al., “Stress Relaxation and Composition of Hydrazone‐Crosslinked Hybrid Biopolymer‐Synthetic Hydrogels Determine Spreading and Secretory Properties of MSCs,” Advanced Healthcare Materials 11 (2022): 2200393. 10.1002/adhm.202200393 PMC9308669 35575970

[42] E. E. Charrier , K. Pogoda , R. G. Wells , and P. A. Janmey , “Control of Cell Morphology and Differentiation by Substrates with Independently Tunable Elasticity and Viscous Dissipation,” Nature Communications 9 (2018): 449. 10.1038/s41467-018-02906-9 PMC5792430 29386514

[43] L. Oliver‐Cervelló , H. Martin‐Gómez , C. Gonzalez‐Garcia , M. Salmeron‐Sanchez , and M. P. Ginebra , “Protease‐Degradable Hydrogels with Multifunctional Biomimetic Peptides for Bone Tissue Engineering,” Frontiers in Bioengineering and Biotechnology 11 (2023): 1192436. 37324414 10.3389/fbioe.2023.1192436 PMC10267393

[44] J. F. M. Verstappen , J. Jin , G. Koçer , et al., “RGD‐functionalized Supported Lipid Bilayers Modulate Pre‐Osteoblast Adherence and Promote Osteogenic Differentiation,” Journal of Biomedical Materials Research Part A 108 (2020): 923–937. 31895490 10.1002/jbm.a.36870

[45] W. Qi , J. Yan , H. Sun , and H. Wang , “Multifunctional Nanocomposite Films for Synergistic Delivery of bFGF and BMP‐2,” ACS Omega 2 (2017): 899–909. 30023619 10.1021/acsomega.6b00420 PMC6044765

[46] O. F. Zouani , C. Chollet , B. Guillotin , and M. C. Durrieu , “Differentiation of Pre‐Osteoblast Cells on Poly(Ethylene Terephthalate) Grafted with RGD and/or BMPs Mimetic Peptides,” Biomaterials 31 (2010): 8245–8253. 20667411 10.1016/j.biomaterials.2010.07.042

[47] L. Fourel , A. Valat , E. Faurobert , et al., “β3 Integrin–Mediated Spreading Induced by Matrix‐Bound BMP‐2 Controls Smad Signaling in a Stiffness‐Independent Manner,” Journal of Cell Biology 212 (2016): 693–706. 26953352 10.1083/jcb.201508018 PMC4792076

[48] V. Fitzpatrick , L. Fourel , O. Destaing , et al., “Signal mingle: Micropatterns of BMP‐2 and fibronectin on soft biopolymeric films regulate myoblast shape and SMAD signaling,” Scientific Reports 7 (2017): 41479. 28134270 10.1038/srep41479 PMC5278375

[49] Q. Wei , A. Holle , J. Li , et al., “BMP‐2 Signaling and Mechanotransduction Synergize to Drive Osteogenic Differentiation via YAP/TAZ,” Advanced Science 7 (2020): 1902931. 32775147 10.1002/advs.201902931 PMC7404154

[50] C. Da Silva Madaleno , J. Jatzlau , and P. Knaus , “BMP Signalling in a Mechanical Context—Implications for Bone Biology,” Bone 137 (2020): 115416. 32422297 10.1016/j.bone.2020.115416

[51] Y.‐K. Wang , X. Yu , D. M. Cohen , et al., “Bone Morphogenetic Protein‐2‐Induced Signaling and Osteogenesis Is Regulated by Cell Shape, RhoA/ROCK, and Cytoskeletal Tension,” Stem Cells and Development 21 (2012): 1176–1186. 21967638 10.1089/scd.2011.0293 PMC3328763

[52] U. Dhawan , H. Jaffery , M. Salmeron‐Sanchez , and M. J. Dalby , “An Ossifying Landscape: Materials and Growth Factor Strategies for Osteogenic Signalling and Bone Regeneration,” Current Opinion in Biotechnology 73 (2022): 355–363. 34735985 10.1016/j.copbio.2021.10.010

[53] P. Zhou , J.‐M. Shi , J.‐E. Song , et al., “Establishing a Deeper Understanding of the Osteogenic Differentiation of Monolayer Cultured Human Pluripotent Stem Cells Using Novel and Detailed Analyses,” Stem Cell Research & Therapy 12 (2021): 41. 33413612 10.1186/s13287-020-02085-9 PMC7792045

[54] T. Katagiri , “Bone Morphogenetic Protein‐2 Converts the Differentiation Pathway of C2C12 Myoblasts Into the Osteoblast Lineage [Published Erratum Appears in J Cell Biol,” The Journal of Cell Biology 127 (1994): 1755–1766. 7798324 10.1083/jcb.127.6.1755 PMC2120318

[55] C. Sieber , J. Kopf , C. Hiepen , and P. Knaus , “Recent Advances in BMP Receptor Signaling,” Cytokine & Growth Factor Reviews 20 (2009): 343–355. 19897402 10.1016/j.cytogfr.2009.10.007

[56] C. S. Hill , “Transcriptional Control by the SMADs,” Cold Spring Harbor Perspectives in Biology 8 (2016): a022079. 27449814 10.1101/cshperspect.a022079 PMC5046698

[57] D. K. Morrison , “MAP Kinase Pathways,” Cold Spring Harbor perspectives in biology 4 (2012): a011254. 23125017 10.1101/cshperspect.a011254 PMC3536342

[58] K. M. Sinha and X. Zhou , “Genetic and Molecular Control of Osterix in Skeletal Formation,” Journal of Cellular Biochemistry 114 (2013): 975–984. 23225263 10.1002/jcb.24439 PMC3725781

[59] J. Guicheux , J. Lemonnier , C. Ghayor , A. Suzuki , G. Palmer , and J. Caverzasio , “Activation of p38 Mitogen‐Activated Protein Kinase and c‐Jun‐NH2‐Terminal Kinase by BMP‐2 and Their Implication in the Stimulation of Osteoblastic Cell Differentiation,” Journal of Bone and Mineral Research 18 (2003): 2060–2068. 14606520 10.1359/jbmr.2003.18.11.2060

[60] J. H. Jun , W.‐J. Yoon , S.‐B. Seo , et al., “BMP2‐activated Erk/MAP Kinase Stabilizes Runx2 by Increasing p300 Levels and Histone Acetyltransferase Activity,” Journal of Biological Chemistry 285 (2010): 36410–36419. 20851880 10.1074/jbc.M110.142307 PMC2978570

[61] S. Gallea , F. Lallemand , A. Atfi , et al., “Activation of Mitogen‐Activated Protein Kinase Cascades is Involved in Regulation of Bone Morphogenetic Protein‐2‐Induced Osteoblast Differentiation in Pluripotent C2C12 Cells,” Bone 28 (2001): 491–498. 11344048 10.1016/s8756-3282(01)00415-x

[62] M. J. Dalby , A. J. García , and M. Salmeron‐Sanchez , “Receptor Control in Mesenchymal Stem Cell Engineering,” Nature Reviews Materials 3 (2018): 1–14.

[63] K. Miyazono , Y. Kamiya , and M. Morikawa , “Bone morphogenetic protein receptors and signal transduction,” Journal of Biochemistry 147 (2010): 35–51. 19762341 10.1093/jb/mvp148

[64] S. Biswas , P. Li , H. Wu , et al., “BMPRIA is Required for Osteogenic Differentiation and RANKL Expression in Adult Bone Marrow Mesenchymal Stromal Cells,” Scientific Reports 8 (2018): 8475. 29855498 10.1038/s41598-018-26820-8 PMC5981611

[65] C. F. Lai and S. L. Cheng , “αvβ Integrins Play an Essential Role in BMP‐2 Induction of Osteoblast Differentiation,” Journal of Bone and Mineral Research 20 (2005): 330–340. 15647827 10.1359/JBMR.041013

[66] A. Nohe , E. Keating , T. M. Underhill , P. Knaus , and N. O. Petersen , “Effect of the Distribution and Clustering of the Type I A BMP Receptor(ALK3) with the Type II BMP Receptor on the Activation of Signalling Pathways,” Journal of Cell Science 116 (2003): 3277–3284. 12829744 10.1242/jcs.00519

[67] A. Guevara‐Garcia , L. Fourel , I. Bourrin‐Reynard , et al., “Integrin‐Based Adhesion Compartmentalizes ALK3 of the BMPRII to Control Cell Adhesion and Migration,” Journal of Cell Biology 221 (2022): 202107110. 10.1083/jcb.202107110 PMC9552562 36205720

[68] J. M. Curran , F. Pu , R. Chen , and J. A. Hunt , “The Use of Dynamic Surface Chemistries to Control Msc Isolation and Function,” Biomaterials 32 (2011): 4753–4760. 21489621 10.1016/j.biomaterials.2011.03.045

[69] W. Kuhlman , I. Taniguchi , L. G. Griffith , and A. M. Mayes , “Interplay Between PEO Tether Length and Ligand Spacing Governs Cell Spreading on RGD‐Modified PMMA‐g‐PEO Comb Copolymers,” Biomacromolecules 8 (2007): 3206–3213. 17877394 10.1021/bm070237o

[70] D. Wu , Y. Hou , Z. Chu , Q. Wei , W. Hong , and Y. Lin , “Ligand Mobility‐Mediated Cell Adhesion and Spreading,” ACS Applied Materials & Interfaces 14 (2022): 12976–12983. 35282676 10.1021/acsami.1c22603

[71] S. J. Attwood , Y. Choi , and Z. Leonenko , “Preparation of DOPC and DPPC Supported Planar Lipid Bilayers for Atomic Force Microscopy and Atomic Force Spectroscopy,” International Journal of Molecular Sciences 14 (2013): 3514–3539. 23389046 10.3390/ijms14023514 PMC3588056

[72] A. Boudaoud , A. Burian , D. Borowska‐Wykret , et al., “FibrilTool, an ImageJ Plug‐in to Quantify Fibrillar Structures in Raw Microscopy Images,” Nature Protocols 9 (2014): 457–463. 24481272 10.1038/nprot.2014.024

[73] L. R. Anderson , T. W. Owens , and M. J. Naylor , “Structural and Mechanical Functions of Integrins,” Biophysical Reviews 6 (2014): 203–213. 28510180 10.1007/s12551-013-0124-0 PMC5418412

[74] V. P. Sharma , B. Tang , Y. Wang , et al., “Live Tumor Imaging Shows Macrophage Induction and TMEM‐Mediated Enrichment of Cancer Stem Cells During Metastatic Dissemination,” Nature Communications 12 (2021): 7300. 10.1038/s41467-021-27308-2 PMC8674234 34911937

[75] W. Rasband and K. Baler . ImageJ Wiki, (2024 Apr 18), https://imagej.net/ij/plugins/image_correlator.html.

