# Supplementary material for: Leveraging Viscosity to Unlock the Osteogenic Potential of BMP‐2 Mimetic DWIVA
Source: Small. 2025 Dec 30;22(11):e08366. doi: 10.1002/smll.202508366 (PMC12921548; doi:10.1002/smll.202508366)

Supporting Information

Leveraging viscosity to unlock the osteogenic potential of BMP-2 mimetic DWIVA

Finlay Cunniffe, Ziyuan Luo, Eva Barcelona-Estaje, Eva Forrest, Manuel Salmeron-Sanchez and Marco Cantini*

**Supplementary Methods**

*Fluorescence recovery after photobleaching (FRAP) – Information on the relative diffusivities of SLBs were obtained through performing FRAP on a Zeiss LSM 980 microscope at cell culture conditions (37°C) to measure the lateral mobility of the bilayers. SLBs were made with fluorescent lipid at a molar concentration of 0.1% and a 10µm (DOPC) or 5µm (DPPC) diameter circular region of interest (ROI) was bleached with a 488 nm laser at 80% intensity for 10 iterations. Five pre-bleach images were obtained to allow for normalisation of the intensity changes over time, and a sufficient number of images were taken to ensure a plateau could be seen in the fluorescence recovery curve for DOPC (this was not possible with DPPC). Analysis of the images was carried using the Fiji Frap Profiler v2 plugin.*(1) *This involved circling a ROI, creating another ROI in an unbleached region nearby and running the plugin (using a single exponential recovery curve). FRAP profiler then plotted the intensity values of both ROIs and normalised the bleach spot to the unbleached region before plotting the fluorescent recovery by fitting it to an exponential model. The resulting half-time of recovery was fitted to a modified equation by Soumpasis* *linking recovery half-time to the diffusivity of a material:*

$$D=0.25\frac{r_{n}^{2}}{t_{1/2}}$$

*where D is the diffusion coefficient, r_n_ is the ROI bleach radius, and t_1/2_ is the half-time of recovery after FRAP.*(2,3)

*Relative fluorescence intensities of surfaces – SLBs and glass surfaces were made and functionalised as before but with FITC-RGD and DWIVA peptides. At least 3 images were taken per well on a Zeiss LSM 980 confocal microscope. Integrated density values for each of the 2% surfaces were used to obtain a mean which was divided by itself to set the baseline; normalised fluorescence values for the 6%-functionalised surfaces were obtained by dividing each integrated density value by the baseline 2% value for the respective condition.*

*Quantification of differentiated cells – The proportion of C2C12 cells expressing either myosin 4 or osteocalcin was assessed using a Fiji macro. Binary masks were generated from the DAPI channel to obtain total cell counts from each image. Then, on the channel of interest, the background was subtracted (rolling ball radius = 50) and a binary mask was generated using the same threshold for each condition (0,40 for myosin 4 and 0,25 for osteocalcin). A new image showing the overlap of these two masks (DAPI and stain of interest) was then generated using the ‘Image Calculator… -> AND’ function. The number of remaining particles over 100 pixels in size were counted and presented as a proportion of the total number of nuclei counted.*

*Distances of BMPRIa/vinculin to cell edge – Multichannel images stained for BMPRIa, vinculin and DAPI were analysed with the use of a Fiji macro, ‘Shortest distance to edge and nucleus’, adapted from* (4)*, which requires a combined stack containing multiple 3-channel images, and a ROI.zip file containing cell outlines. Cell outlines were obtained by generating a binary mask from the vinculin channel after applying ‘despeckle’ and ‘Gaussian blue’ functions. The macro saved each of the cell outlines separately alongside the corresponding nuclear outline obtained after applying a binary mask to DAPI-stained images. Each of the vinculin or BMPRIa objects within a cell were then obtained using the following method: 1) selecting the relevant channel, 2) subtracting the background (rolling ball radius = 50), 3) applying a binary mask (with a set threshold of 0,6167 for vinculin or 0,10000 for BMPRIa), and 4) using the ‘analyse particles’ function to select the objects and then save them as a multipoint ROI. A minimum pixel size of 5 was used for BMPRIa, while vinculin objects were filtered to only include objects with a major axis of ≥ 1μm. For each object, the shortest distance to both the cell outline and the nuclear outline was measured (objects located outside the cell or inside the nucleus were excluded). Normalised distances from the cell edge represent the distance to the cell edge as a proportion of the total distance to both the edge and nucleus, with 0 being next to the cell edge and 1 being at the nucleus.*

*BMPRIa/vinculin colocalisation - Multichannel images stained for BMPRIa, vinculin and DAPI were analysed with the use of a Fiji macro, ‘Shortest distance to other objects’, adapted from* (4)*, which requires a combined stack containing multiple 3-channel images, and a ROI.zip file containing cell outlines. Cell outlines were obtained by generating a binary mask from the vinculin channel after applying ‘despeckle’ and ‘Gaussian blue’ functions. Each of the vinculin and BMPRIa objects within a cell were then obtained using the following method: 1) selecting the relevant channel, 2) subtracting the background (rolling ball radius = 50), 3) applying a binary mask (with a set threshold of 0,6167 for vinculin or 0,10000 for BMPRIa), and 4) using the ‘analyse particles’ function to select objects larger than 5 pixels. For BMPRIa, the centroid of each object was saved as a multipoint ROI, while for vinculin each shape was added as a separate object in the ROI manager and saved all together. The distance of each BMPRIa object to the nearest vinculin object was measured iteratively, with a distance of 0 μm corresponding to BMPRIa objects found to be overlapping with vinculin.*

*References:*

1. The Hardin Lab. The Hardin Lab. 2018 [cited 2023 Apr 26]. Microscopy. Available from: http://worms.zoology.wisc.edu/Library/WebServer/Documents/research/4d/4d.html

2. Soumpasis DM. Theoretical analysis of fluorescence photobleaching recovery experiments. Biophys J. 1983 Jan 1;41(1):95–7.

3. Kang M, Day CA, Kenworthy AK, DiBenedetto E. Simplified Equation to Extract Diffusion Coefficients from Confocal FRAP Data. Traffic. 2012;13(12):1589–600.

4. Sharma VP, Tang B, Wang Y, Duran CL, Karagiannis GS, Xue EA, et al. Live tumor imaging shows macrophage induction and TMEM-mediated enrichment of cancer stem cells during metastatic dissemination. Nat Commun. 2021 Dec 15;12(1):7300.

Supplementary Figures

Figure S1: DOPC and DPPC bilayers have different diffusivities and RGD distribution unaffected by addition of DWIVA


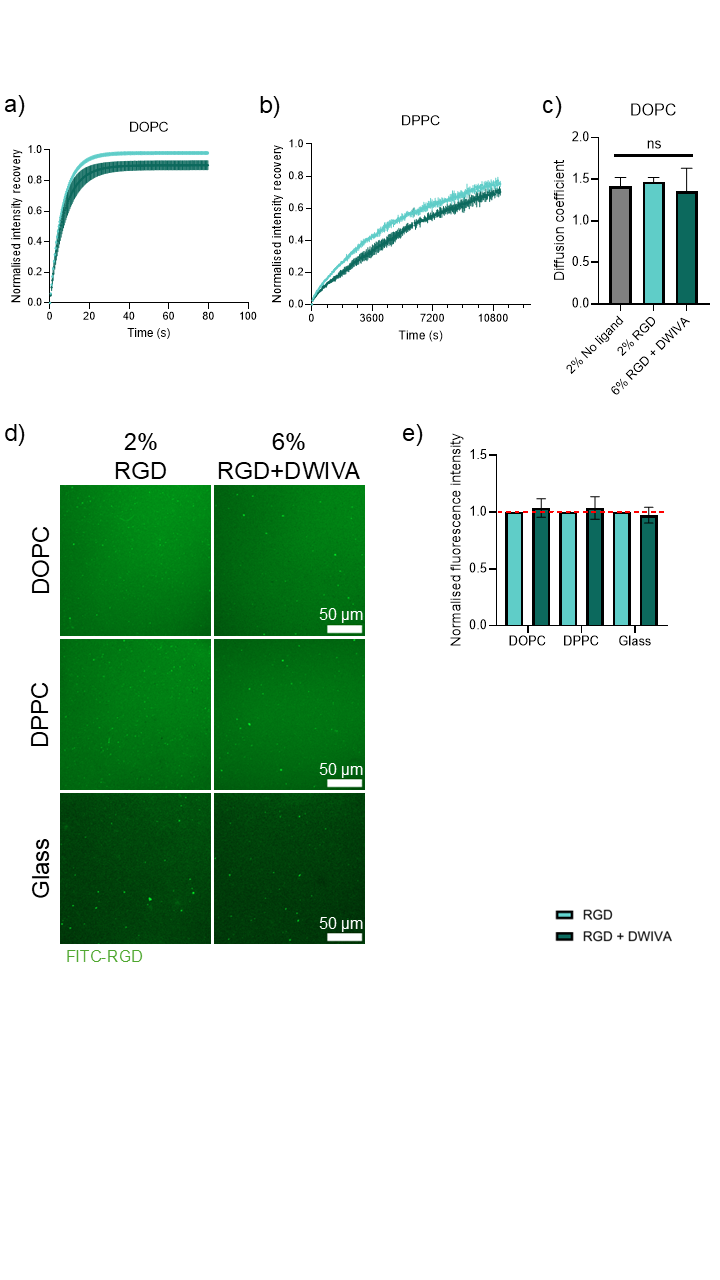


Figure S1: a-c) Relative diffusivities of supported lipid bilayers (SLBs) made through vesicle fusion with either 2% or 6% biotinylated lipid for functionalisation with RGD or RGD+DWIVA, respectively. 0.1% TopFlour-PE was added for visualisation of the bilayers. Fluorescence Recovery After Photobleaching (FRAP) recovery curves were obtained for SLBs made with either a) DOPC (n = 9) or b) DPPC (n = 3). c) The half-time of recovery for DOPC (n = 9) showed that the diffusion coefficient for DOPC SLBs remained unchanged with different amounts of biotinylated lipid and ligands. In all graphs n represents individual bleach regions. In graph c) data represented as means ± SD and statistical significance was determined using D’Agostino Pearson normality test, followed by an ordinary one-way ANOVA with Holm-Šidák. ns = p>0.05. d-e) Relative fluorescent intensity of surfaces functionalised with FITC-conjugated RGD ligands. d) Representative images of each surface; and e) quantification of integrated density for each of the 6% substrates normalised to the mean integrated density from 2% surfaces for each condition (n=16, 16, 12 for DOPC, DPPC and glass, with integrated density values obtained from images across n = 3 wells).

Figure S2: Cells require adhesive ligand RGD to bind to the SLBs even in the presence of DWIVA


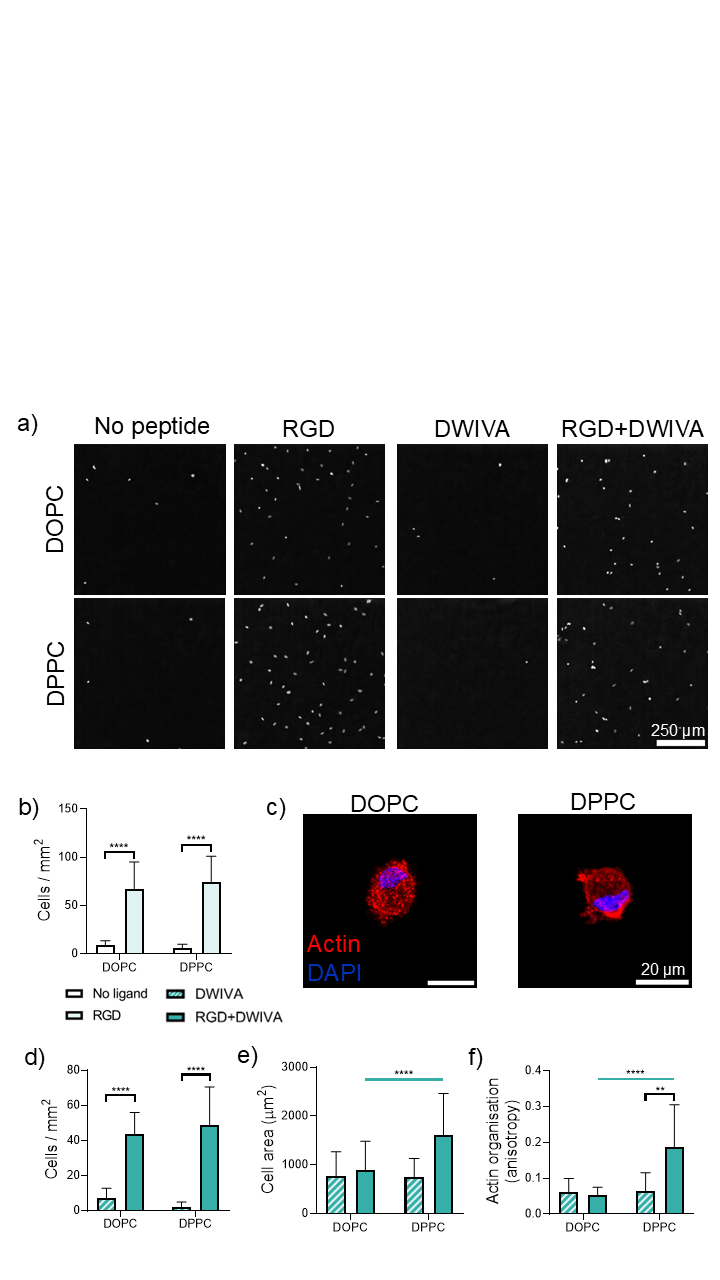


Figure S2: a) Representative images of hMSC nuclei after 24-hour culture on SLBs, stained for DAPI (scale bar = 250 µm). b) Quantification of cell density on SLBs after seeding at a density of 100 cells/mm^2^ show the SLBs to be non-fouling surfaces in the absence of RGD (n=10, 15, 15, 15). c) Representative images of the few cells present on DOPC and DPPC SLBs functionalised with only DWIVA and no RGD, stained for actin (red) and DAPI (blue). d-f) Quantification of hMSC morphological response after 24-hour culture show RGD is necessary to observe cell response to DWIVA on the bilayers: d) Cell density (n=15 for all conditions); e) cell area (n=8, 63, 4, 53); and f) Organisation of actin cytoskeletal fibres (n=22, 26, 13, 24). Data represented as means ± SD. In graphs b+d) n represents frames of DAPI stained nuclei; in e) n represents cells; in f) n represents individual regions of the cytoskeleton, with 3-4 measured per cell. Statistical significance was determined using D’Agostino Pearson normality test, followed by an ordinary two-way ANOVA with Tukey’s multiple comparison test for b+d), and a Kruskal-Wallis with Dunn’s multiple comparison test for e-f). * p<0.05, ** p<0.01, *** p<0.001, **** p<0.0001.

Figure S3: Stability of SLBs and reorganization of functional layer by cells

**
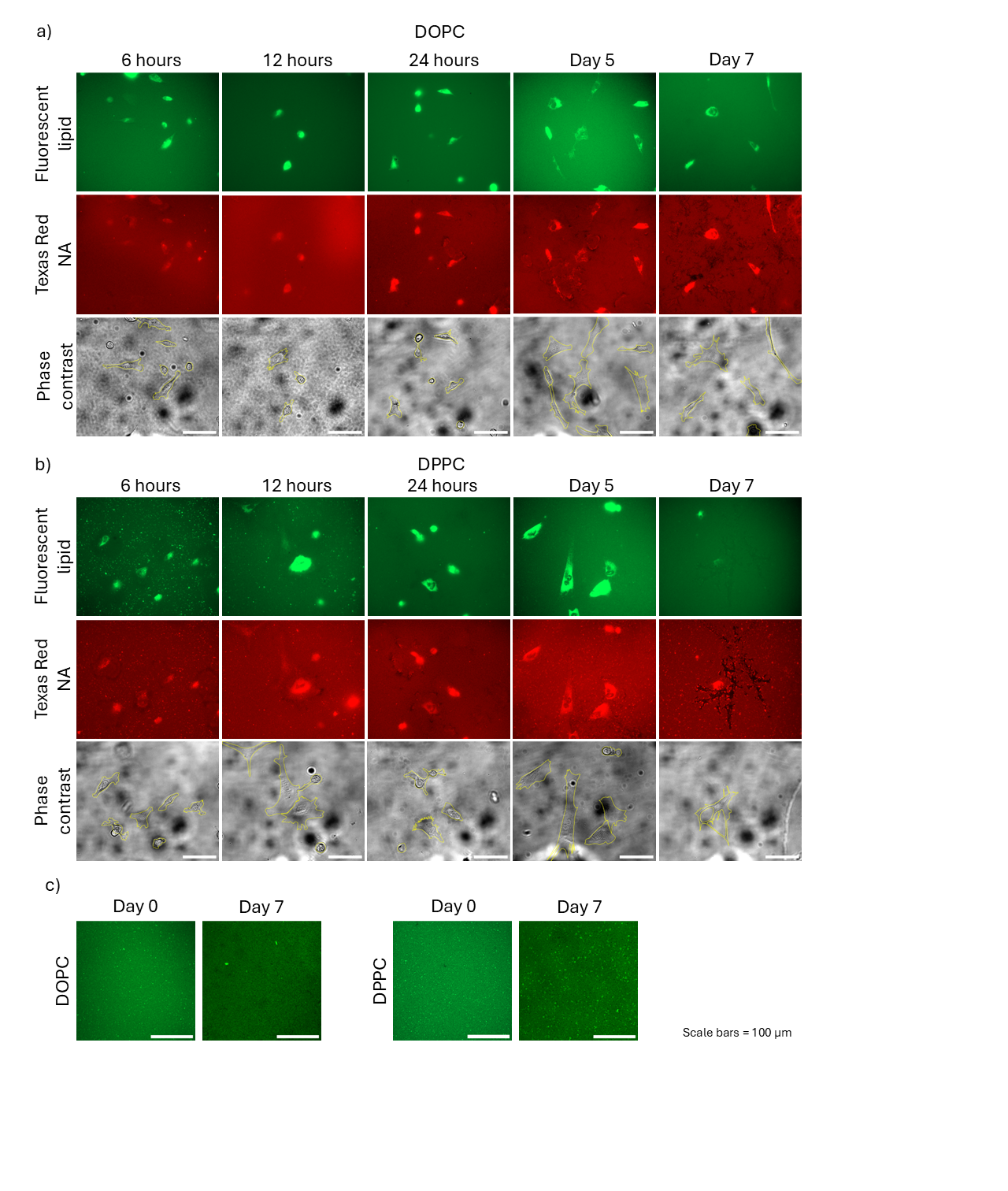
**

Figure 3: a-b) MSCs cultured on a) DOPC and b) DPPC SLBs made with 0.1% fluorescent lipid and 0.02 mg/ml Texas Red NeutrAvidin for visualisation of SLB integrity and cell deformation kinetics of functional layers. SLBs were made with 6% biotin-lipid and functionalised with both RGD and DWIVA. c) High resolution confocal images showing stability of SLBs in the absence of cell seeding. Scale bars = 100 µm.

**Figure S4: C2C12 cells exhibit viscosity-dependent changes in cell morphology that are unaffected by DWIVA functionalisation**


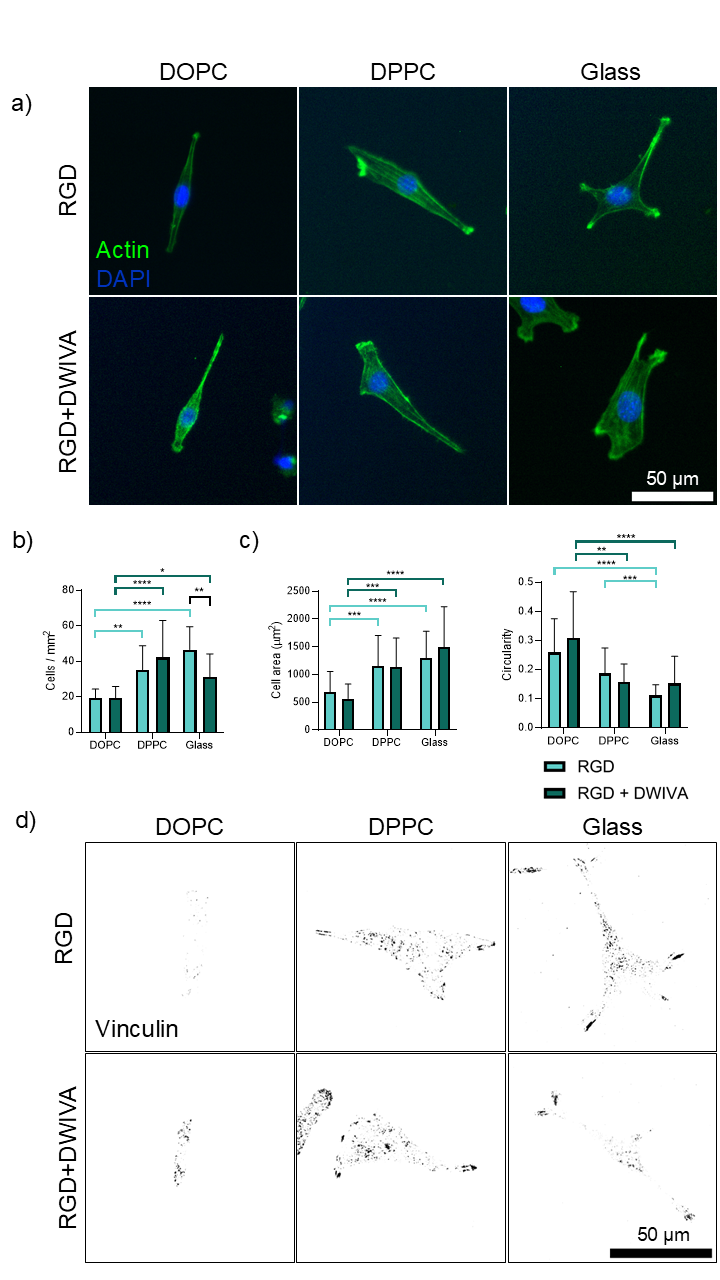


Figure S4: a) Representative images of C2C12 myoblast cells after 24-hours culture on SLBs and non-mobile glass, stained for actin (green) and DAPI (blue) (scale bar = 50 µm). b-c) Quantification of C2C12 cell morphological response after 24-hour culture: b) Cell density (n=15 in all conditions); c) Cell area (n=36, 19, 41, 44, 48, 45) and cell circularity (n=36, 19, 41, 44, 48, 45). d) Representative images of C2C12 cells stained with vinculin after 24-hour culture, with a threshold and binary mask applied to visualise focal adhesions (scale bar = 50 µm). Data represented as means ± SD. In graph b) n represents frames of DAPI stained nuclei; in graphs c) n represents cells. Statistical significance was determined using D’Agostino Pearson normality test, followed by an ordinary two-way ANOVA with Tukey’s multiple comparison test for b), and a Kruskal-Wallis with Dunn’s multiple comparison test for c). * p<0.05, ** p<0.01, *** p<0.001, **** p<0.0001.

Figure S5: Focal adhesion size and impact of differing BMP-2 concentrations


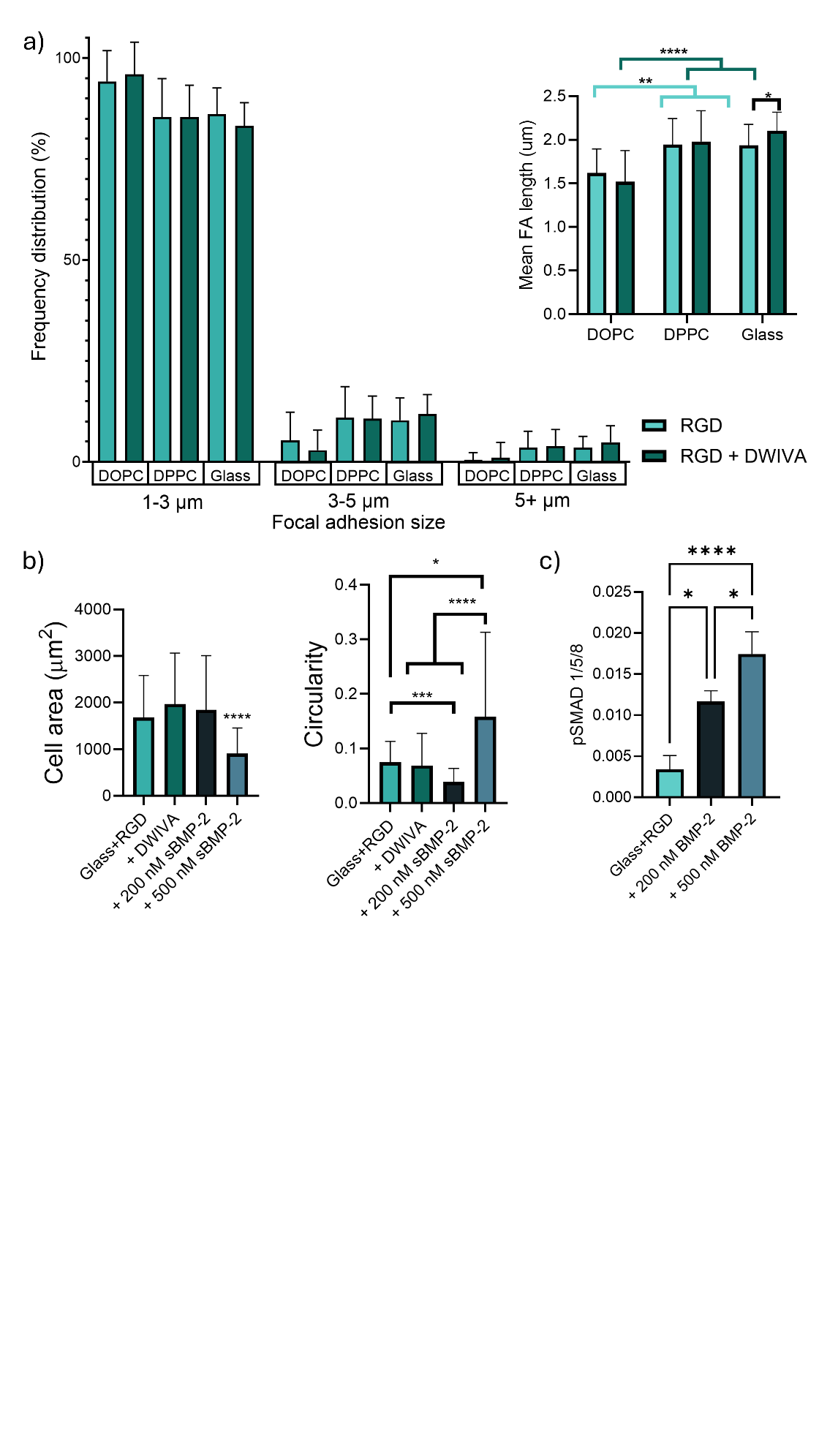


Figure S5: a) Further quantifications of focal adhesion size after applying threshold and binary mask to vinculin-stained images: Frequency distribution of focal adhesion sizes and mean focal adhesion length (n=26, 26, 26, 28, 28, 25). b) Analysis of hMSC cell morphology after 24-hour culture on non-mobile glass with different ligand functionalisation or addition of soluble-BMP-2 (n=89, 82, 65, 82). c) Canonical signalling protein phospho-SMAD 1/5/8 after 90 minutes of culture (n=9 in all conditions) as measured by in-cell western (n=9 in all conditions). Data represented as means ± SD. In graphs a-b) n represents individual cells; in graph c) n represents regions of interest drawn on n=3 wells. Statistical significance was determined using D’Agostino Pearson normality test, followed by a Kruskal-Wallis with Dunn’s multiple comparison test. * p<0.05, ** p<0.01, *** p<0.001, **** p<0.0001.

Figure S6: Osterix staining of MSCs


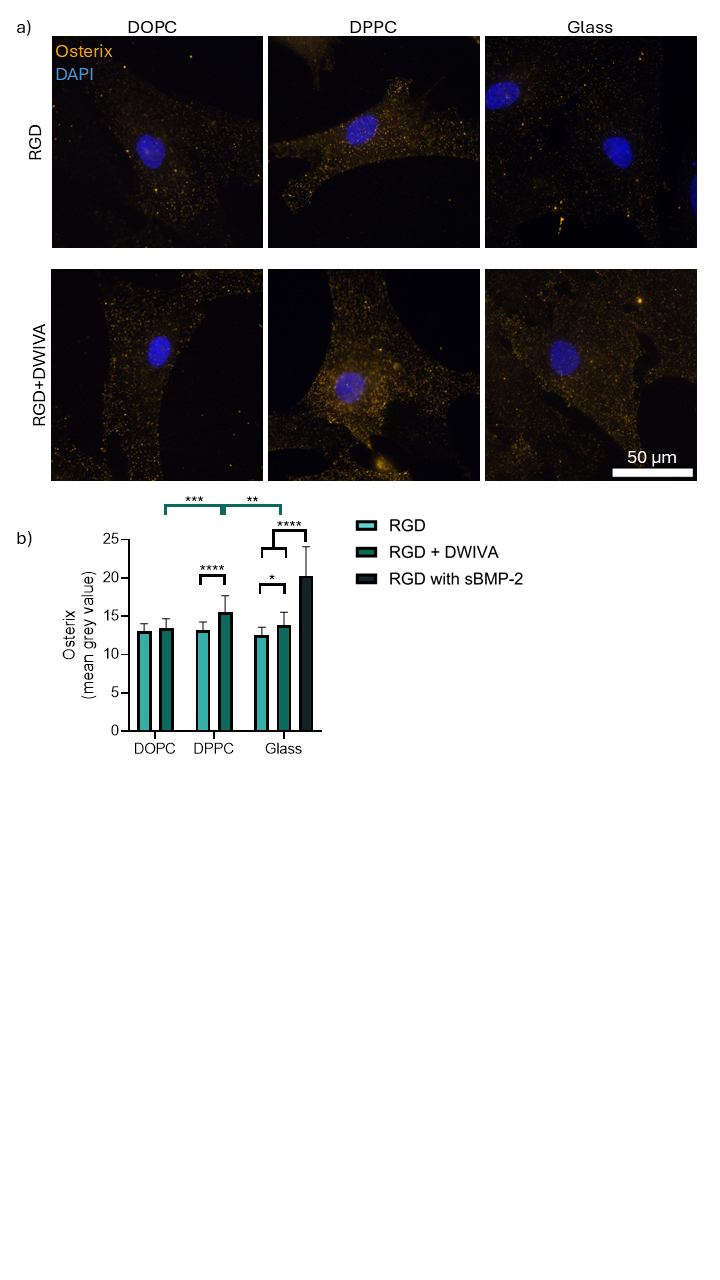


Figure S6: a) Representative images of hMSCs after 7-day culture on SLBs and non-mobile glass, stained for osterix (yellow) and DAPI (blue) (scale bar = 50 µm). b) Quantification of osterix mean grey value in MSCs after 7-days (n=15,14,13,14,13,12,13). Data represented as means ± SD. In all graphs n represents individual cells across n=3 wells. Statistical significance was determined using D’Agostino Pearson normality test, followed by an ordinary two-way ANOVA with Tukey’s multiple comparison test, and an ordinary one-way ANOVA with Holm-Šidák for the glass controls. * p<0.05, ** p<0.01, *** p<0.001, **** p<0.0001.

Figure S7: Myogenic differentiation of C2C12 cells


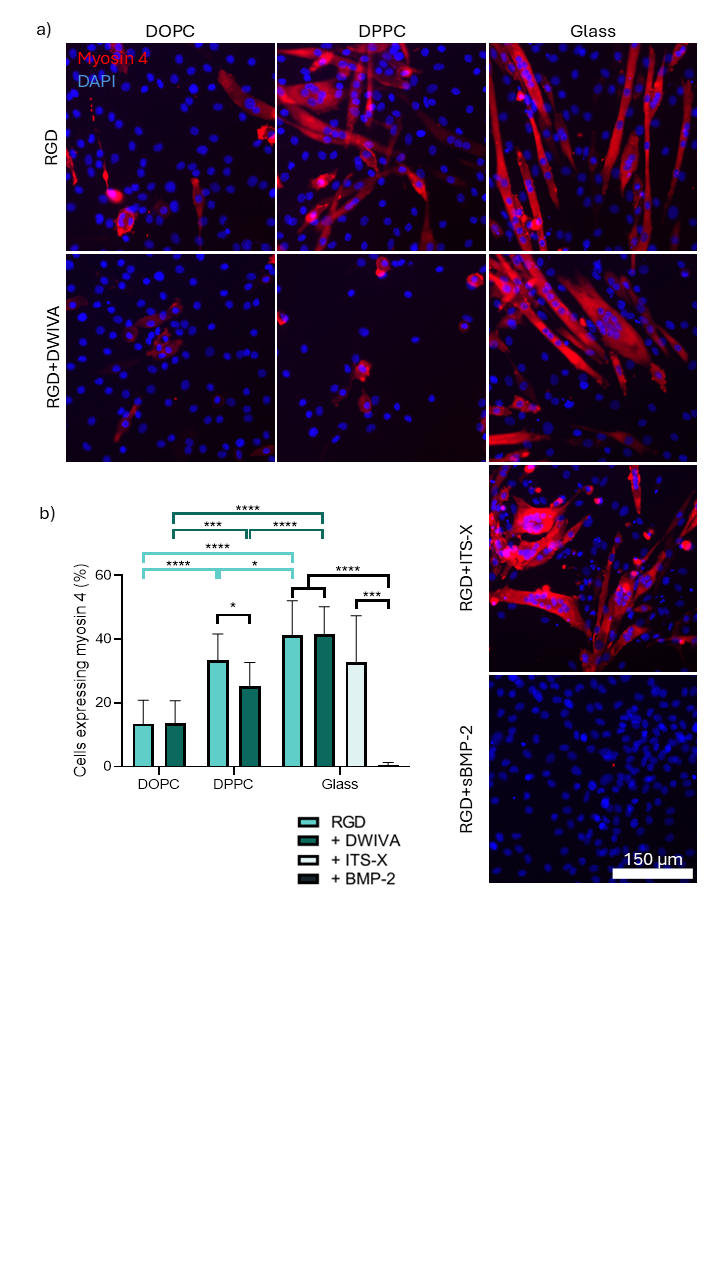


Figure S7: Differentiation of C2C12 myoblasts after 5-day culture on SLBs and non-mobile glass, functionalised with RGD ± DWIVA ligands. Glass+RGD also had either soluble ITS-X (myogenic control) or soluble BMP-2 (osteogenic control) added. a) Representative images of cells stained for Myosin 4 (red) and DAPI (blue) (scale bar = 150 µm). b) Quantification of cells expressing myosin 4 after thresholding and creating a binary mask of the images based on the myogenic stain (n=15 in all conditions). Data represented as means ± SD and n represents images containing multiple cells across n=3 wells. Statistical significance was determined using D’Agostino Pearson normality test, followed by an ordinary two-way ANOVA with Tukey’s multiple comparison test, and an ordinary one-way ANOVA with Holm-Šidák for the glass controls. * p<0.05, ** p<0.01, *** p<0.001, **** p<0.0001.

Figure S8: Osteogenic differentiation of C2C12 cells


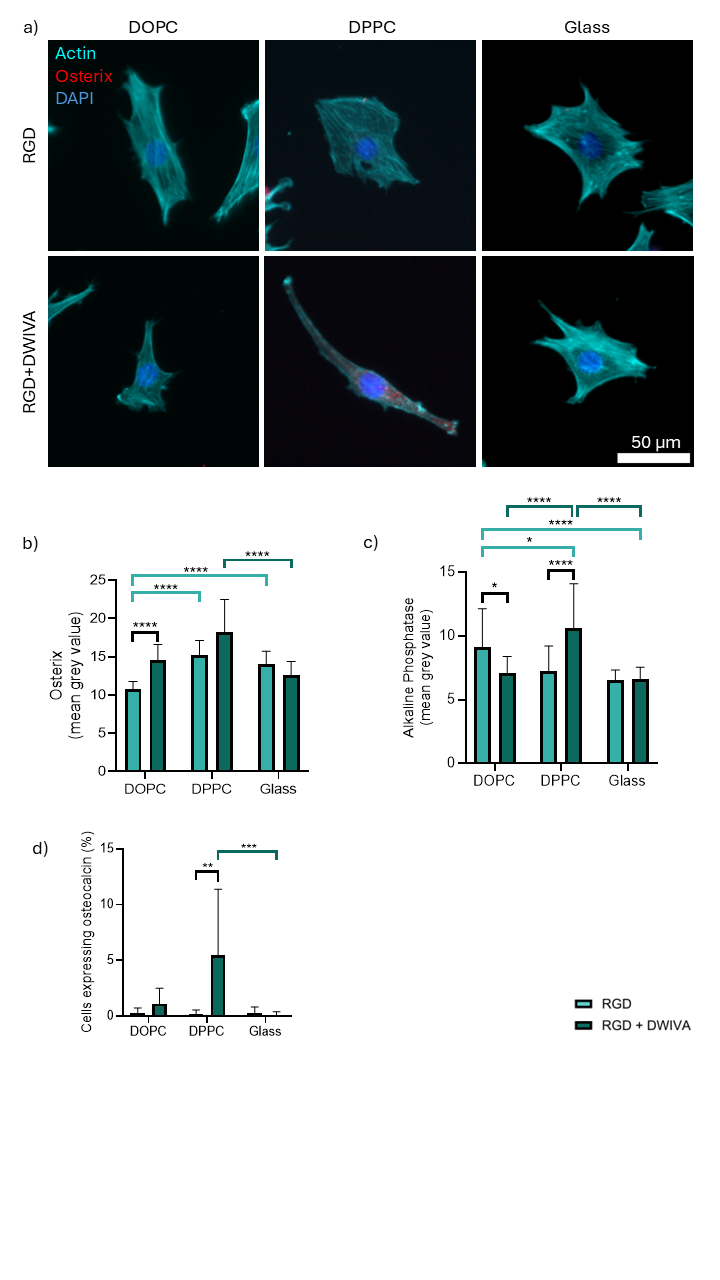


Figure S8: Expression of differentiation markers in C2C12 cells cultured on SLBs and non-mobile glass, functionalised with RGD ± DWIVA ligands. Representative images of C2C12 cells. a) Representative images of cells stained for osterix (red), actin (cyan) and DAPI (blue) after 3 days of culture (scale bar = 50 µm). b) Quantification of osterix mean grey value in cells after 3 days of culture (n=21,22,26,29,32,32). c) Quantification of Alkaline phosphatase mean grey value in cells after 5 days of culture (n=28,31,41,44,31,27). d) Quantification of cells expressing osteocalcin after 8 days of culture, after thresholding and creating a binary mask of images based on the osteogenic stain (n=15 in all conditions). Data represented as means ± SD. In graphs b-c) n represents individual cells; in graph d) n represents images containing multiple cells across n=3 wells. Statistical significance was determined using D’Agostino Pearson normality test, followed by a Kruskal-Wallis with Dunn’s multiple comparison test. * p<0.05, ** p<0.01, *** p<0.001, **** p<0.0001.

Figure S9: Summary of non-canonical BMP-2 signalling pathways


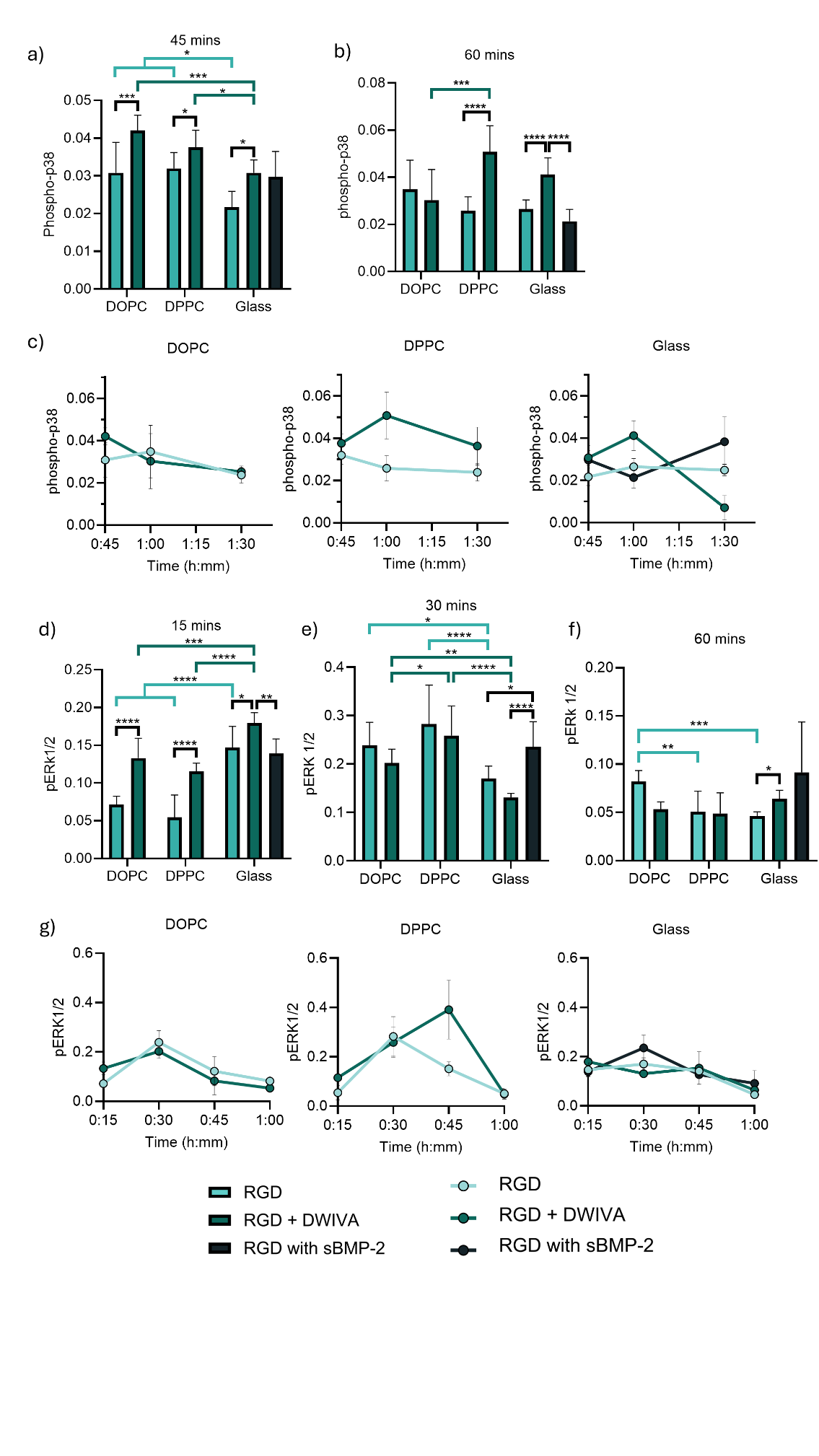


Figure S9: Signalling of non-canonical BMP-2 pathways on SLBs and non-mobile glass controls functionalised with RGD ± DWIVA ligands. Soluble BMP-2 added on glass at 200 ng/ml as a control. Phospho-p38 measured by in-cell western after a) 45 minutes (n=9,6,9,9,3,9,9) and b) 60 minutes (n=9 in all conditions) of culture; c) shows a summary of each time point. pERK1/2 was measured by in-cell western after d) 15 minutes (n=9 in all conditions), e) 30 minutes (n=9 in all conditions) and f) 60 minutes (n=9,9,9,9,9,9,6) of culture; g) shows a summary of each time point looked at for pERK1/2. Data represented as means ± SD, and in all graphs n represents regions of interest drawn on n=3 wells (in graph a) this is across n=1-3 wells). Statistical significance was determined using D’Agostino Pearson normality test, followed by a two-way ANOVA with Tukey’s multiple comparison test for a-e) and a Kruskal-Wallis with Dunn’s multiple comparison test for f). Glass controls were assessed with a Kruskal-Wallis for a+e), and an ordinary one-way ANOVA for b+d). * p<0.05, ** p<0.01, *** p<0.001, **** p<0.0001.

Figure S10: Representative images of vinculin/BMPRIa staining


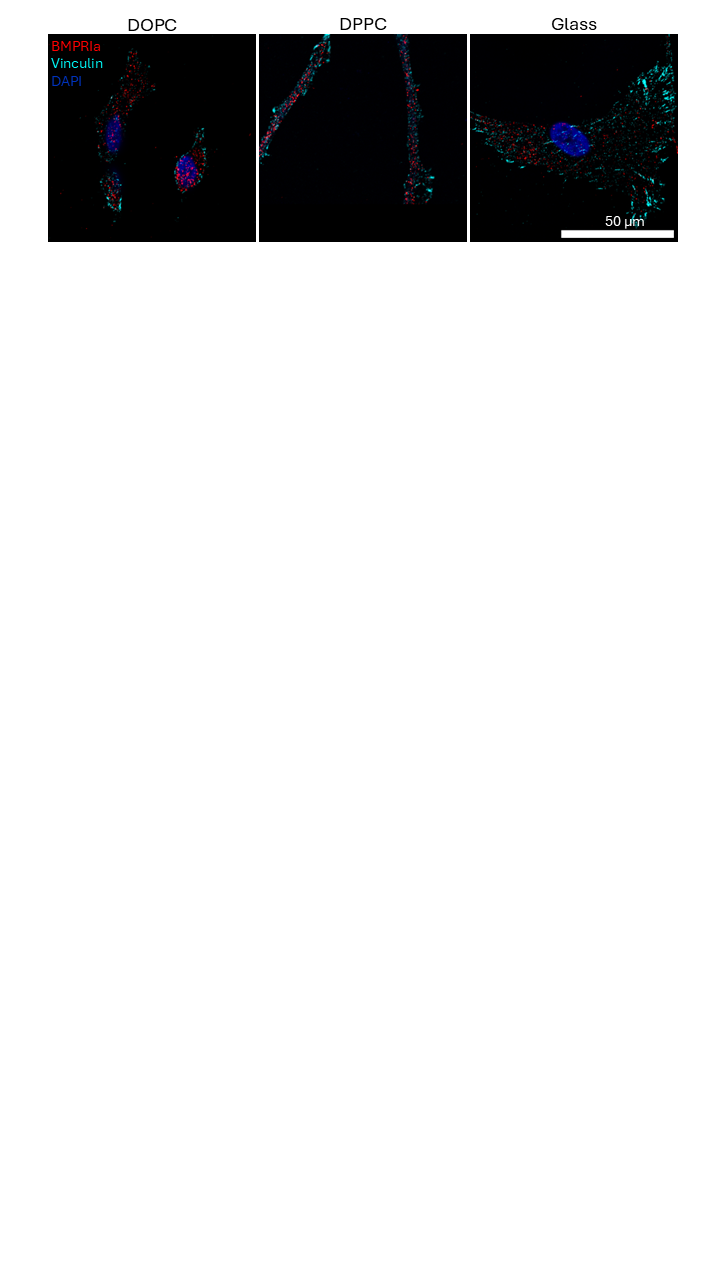


Figure S10: Representative images showing hMSCs stained for BMPRIa (red), vinculin (cyan) and DAPI (blue) on surfaces functionalised with only RGD ligands, after 24 hours of culture (scale bar = 50 μm).

Figure S11: Integrin staining


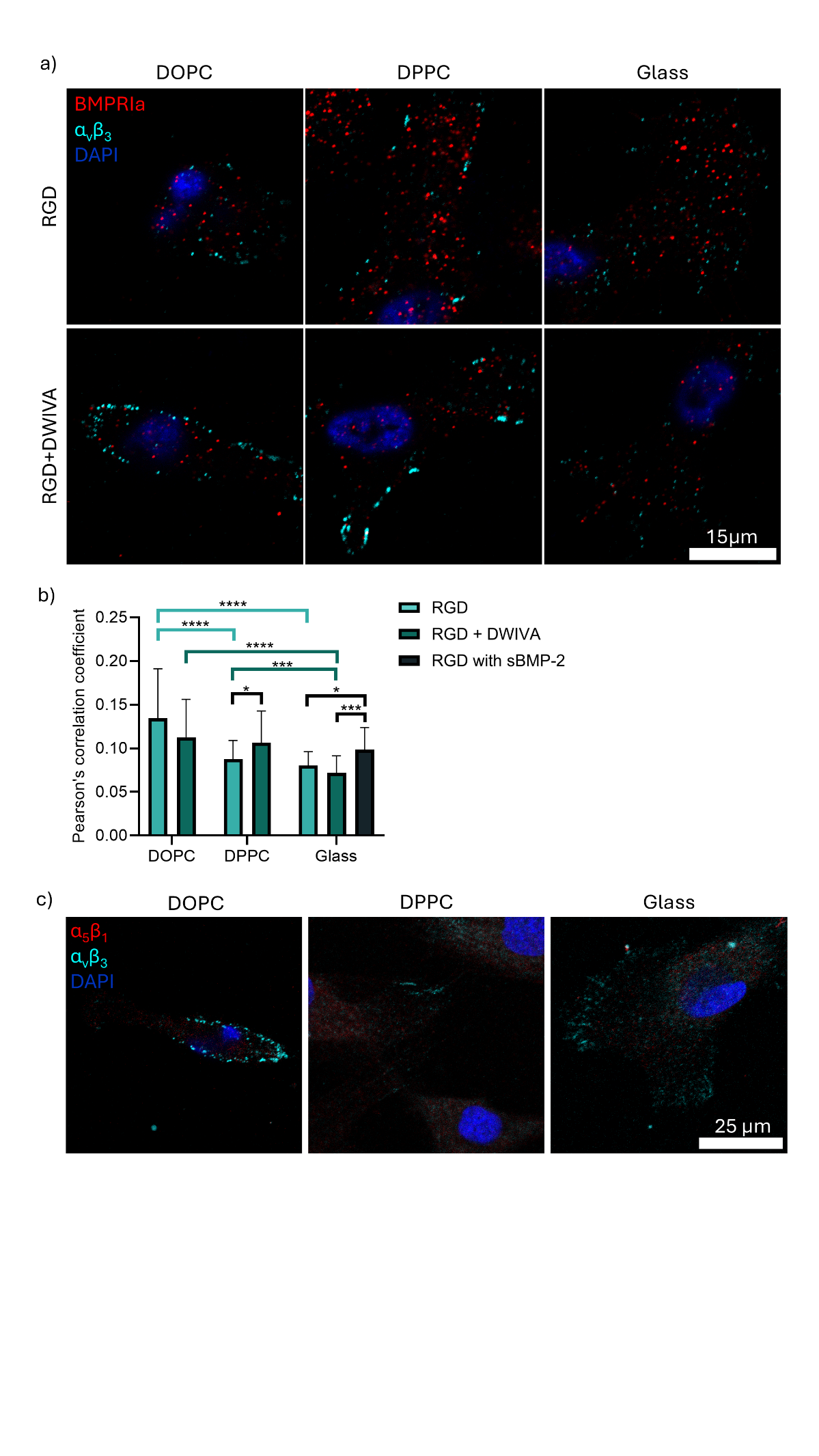


Figure S11: a) Representative images showing hMSCs stained for BMPRIa (red), integrin α_v_β_3_ (cyan) and DAPI (blue) on surfaces functionalised with only RGD ± DWIVA ligands, after 24 hours of culture (scale bar = 15 μm). b) Pearson’s Correlation Coefficient of BMPRIa and integrin α_v_β_3_ at regions of the cell edge (n=27,27,29,27,27,30,29). c) Representative images showing hMSCs stained for integrins α_5_β_1_ (red), α_v_β_3_ (cyan) and DAPI (blue), on surfaces functionalised with only RGD ± DWIVA ligands after 24 hours of culture (scale bar = 25 μm). Data represented as means ± SD and n represents edge regions from individual cells. Statistical significance was determined using D’Agostino Pearson normality test, followed by a Kruskal-Wallis with Dunn’s multiple comparison test. * p<0.05, ** p<0.01, *** p<0.001, **** p<0.0001.

Table S1: Percentage moles of biotinylated lipid (b-cap-PE) added when functionalizing with different combinations of ligands.

| Lipid | MW (g/mol) | Functional lipids (%) | | |
| --- | --- | --- | --- | --- |
|  |  | RGD-only | DWIVA-only | RGD+DWIVA |
| DOPC | 786.112 | 2 | 4 | 6 |
| DPPC | 734.039 | 1.86 | 3.73 | 5.60 |

Figure S12: PDMS shapes created to house substrates and cells on a 30 mm coverslip.
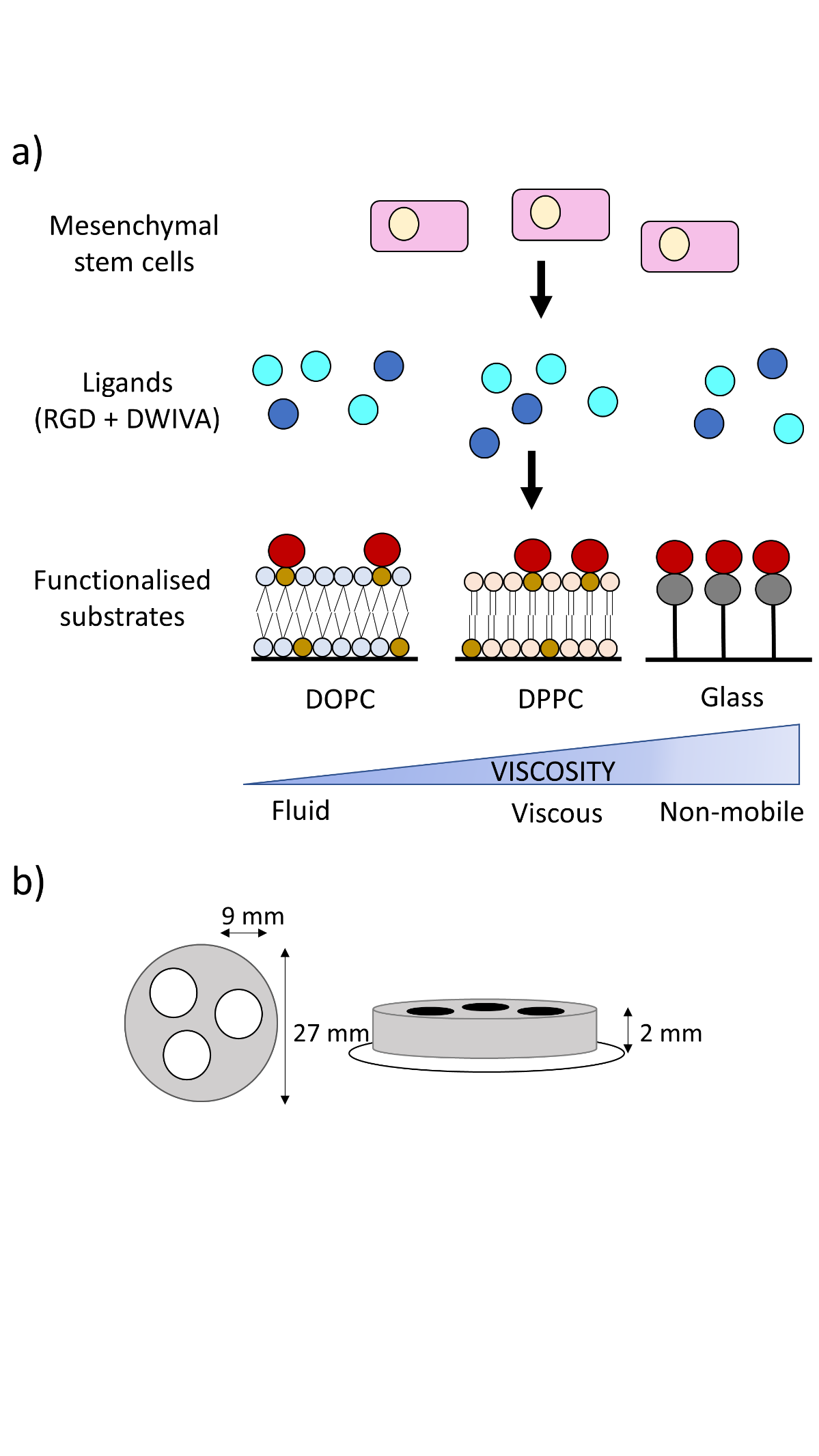


Figure S13: Polygonal shapes drawn on actin cytoskeleton images to analyse anisotropy (organization) of actin fibres.
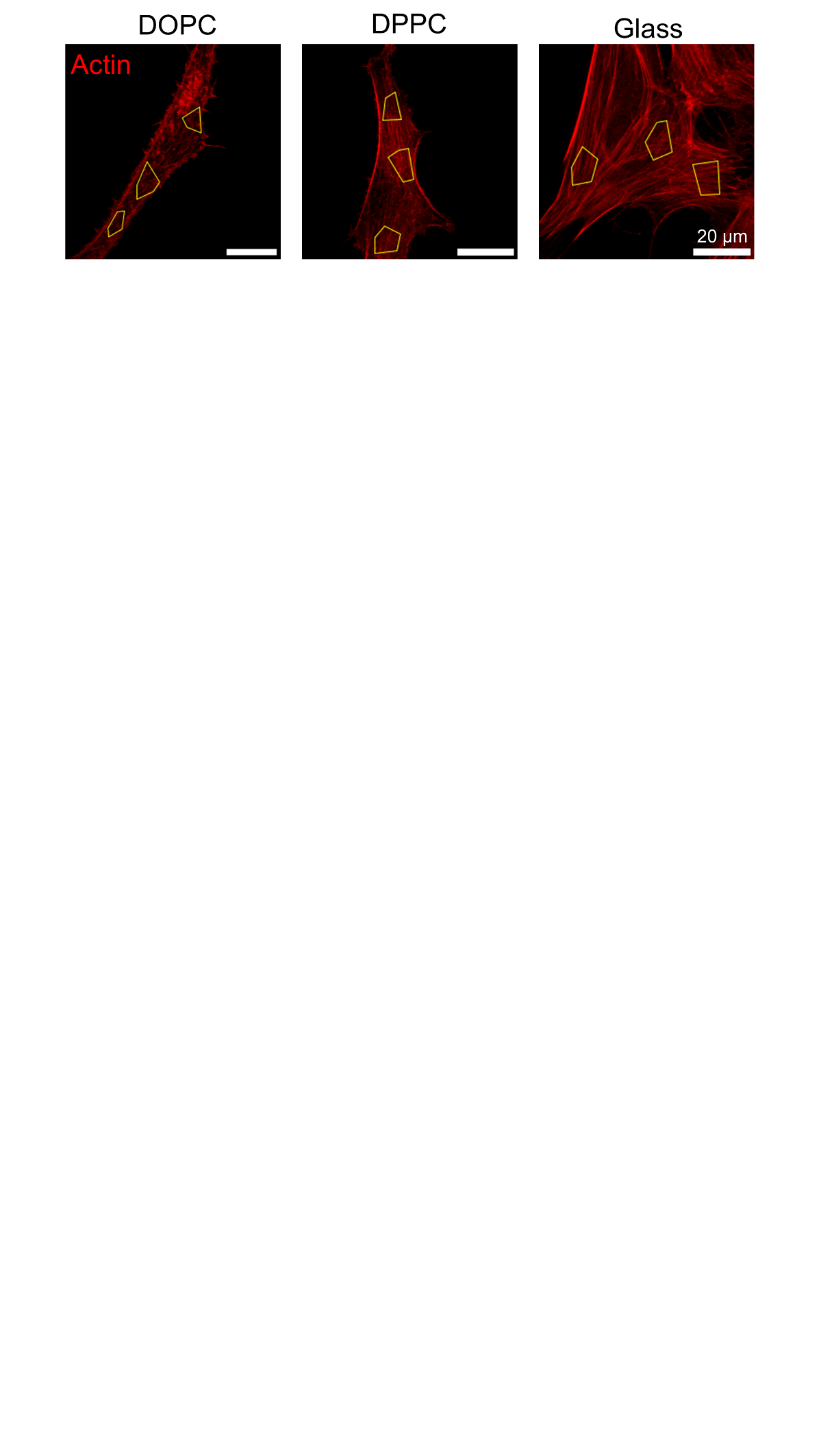

Supplement: Supplementary file 1 — Supporting File: smll72188‐sup‐0001‐SuppMat.docx [file SMLL-22-e08366-s001.docx]
